# Automated eDNA and eRNA profiling for biodiversity monitoring in marine and freshwater ecosystems

**DOI:** 10.1038/s41598-026-58421-1

**Published:** 2026-06-25

**Authors:** Robert G. Beiko, Jennifer Tolman, Soma Sardar Barawi, Mohamed Fares, Sneha Surya Narayana Murthy, Tom Knox, Connor M. Mackie, Iain Grundke, Nicholas W. Jeffery, Ryan R. E. Stanley, Vincent Sieben, Julie LaRoche

**Affiliations:** 1 Faculty of Computer Science, Dalhousie University, Halifax, Nova Scotia Canada; 2 Institute for Comparative Genomics, Dalhousie University, Halifax, Nova Scotia Canada; 3 Dartmouth Ocean Technologies, Inc., Dartmouth, Nova Scotia Canada; 4 Department of Biology, Dalhousie University, Halifax, Nova Scotia Canada; 5 Coastal Ecosystem Science Division, Fisheries and Oceans Canada, Dartmouth Nova Scotia, Canada; 6 Department of Electrical and Computer Engineering, Dalhousie University, Halifax Nova Scotia, Canada

**Keywords:** Environmental DNA, Environmental RNA, Automated eDNA sampling, Aquatic habitats, Metagenomics, Ecology, Ecology, Environmental sciences, Microbiology, Ocean sciences

## Abstract

Biodiversity monitoring is essential to measure the impacts of pollution, invasive species, and the longer-term effects of climate change. Automated samplers enable temporally flexible, remote collection of environmental DNA (eDNA), improving access to time-sensitive events. The Dartmouth Ocean Technologies (DOT) Preserving eDNA Sampler has proven effective in multi-month marine deployments, but further validation is needed across a broader range of habitats and water chemistries, and to establish its suitability for collection and assessment of environmental RNA (eRNA). In this study, we collected samples near the surface (1–1.5 m depth) of a brackish pond, a freshwater lake, and two marine harbours. We identified patterns of species turnover consistent with transitions among aquatic environments, including invasive species such as smallmouth bass and chain pickerel in the freshwater lake. Automated deployment in Halifax Harbour following a significant rainfall event detected nearly ten times as many probable fecal-associated bacteria by proportion at this site relative to Lunenburg Harbour. Preserved eRNA allowed the identification of taxa below the eDNA limit of detection. Our pilot study demonstrates the feasibility of using the DOT sampler for longer-term biomonitoring in a diverse range of aquatic habitats, yielding ecological insights that would not be attainable through manual sampling alone.

**Supplementary Information:**

The online version contains supplementary material available at https://doi.org/10.1038/s41598-026-58421-1.

## Introduction

Traditional biomonitoring in aquatic environments has relied on a wide range of methods tailored to specific environmental and logistical constraints, such as sampling depth and site accessibility. Environmental DNA (eDNA) can augment or replace traditional methods in aquatic settings, due to its sensitivity, its cost effectiveness, its non-invasive nature, and the potential to standardize biodiversity sampling across habitats (e.g., Deiner et al.,^1^, He et al.,^2^, Picq et al.,^3^). eDNA can identify living and dead organisms depending on shedding rate and DNA stability for a given set of environmental factors^4–6^.

Remote or extreme environments such as the deep sea or polar regions present significant sampling challenges, often due to limited resources or physical constraints of repeated sampling in situ^7^. Autonomous eDNA samplers can reduce the need for costly and potentially risky travel to remote locations (e.g., Cote^8^). Several commercial- and research-grade eDNA samplers are available to end users^9^ that differ by degree of autonomy, depth tolerance, prevention of sample cross contamination, filter pore size, and sample preservation, including the Robotic Cartridge Sampling Instrument (RoCSI) developed by the National Oceanographic Center (Southampton, UK); the Ocean Diagnostics Inc. Ascension; and the Smith-Root autosampler (SR-eDNA^10^).

Once retrieved, automated samples can be analyzed using techniques that include amplicon analysis to provide a detailed profile of biodiversity, and metagenomic techniques that provide information about microbial pathways and other functions^11–14^. Environmental RNA (eRNA) is emerging as a tool to characterize living organisms, contemporary community structure, and magnitude of in situ gene expression responses to stressors^15,16^. The instability of eRNA complicates its analysis^17^; preservation offers an opportunity to stabilize eRNA samples for later analysis. However, few studies to date have reported eRNA recovery from automated samplers.

The Dartmouth Ocean Technologies Inc. (DOT) Preserving eDNA sampler^18^ has a capacity for nine filters with different pore-size options, is fully programmable, has sample preservation to support multi-month deployments, and uniquely uses acid cleaning between samples to prevent cross-contamination. During a nine-week test deployment in Halifax Harbour, three pre-programmed DOT samplers detected comparable marine fish diversity to water samples collected each week filtered on a standard vacuum pump and showed minimal biofouling on the water inlet and outlet ports^19^. DNA concentrations preserved in situ by the DOT sampler showed no evidence of DNA degradation over nine weeks, comparable to preserving the fresh sample filters at −80 °C. Beyond ground truthing with manual samples, Hendricks et al.,^18^ demonstrated high concordance in prokaryotic and eukaryotic profiles from samples collected using the DOT sampler and manual Niskin bottles in a transect in Bedford Basin, Nova Scotia. The authors of this study highlighted the need to perform tests in a wider range of aquatic environments, and to examine the ability of the sampler to collect and preserve eRNA.

The province of Nova Scotia, Canada, is a highly biodiverse environment due in part to an extensive variety of ocean, coastal, and freshwater habitats. The ocean is a key driver of the provincial economy through fishing, tourism, and industrial activities such as resource extraction and shipbuilding. Species including the American eel (Katew), eelgrass (Qata’skwl), and lobster (Jakej) are culturally important to Indigenous Mi’kmaq communities^20^. eDNA-based efforts to monitor biodiversity in Nova Scotia include the 13-year microbial Bedford Basin monitoring program^21^, eDNA-based assessment of eelgrass distribution and diversity^22,23^, and the assessment of invasive species including bryozoans and tunicates using qPCR^24^. The temporal and spatial depth of these and other biomonitoring efforts can be augmented by automated sample collection. To demonstrate its fitness for purpose, here we deploy a DOT sampler in multiple aquatic settings and analyze preserved eDNA and eRNA. Deployments were carried out in a brackish pond, a freshwater lake, and two marine harbours in Nova Scotia. In each case, we profiled prokaryote, eukaryote, and fish diversity using standard genetic markers, and used the results to address specific questions about native and invasive species, and evidence of contamination.

## Methods

### The DOT eDNA sampler

The DOT eDNA sampler is an autonomous sampling system capable of long-term deployments in remote locations as described in Hendricks et al.,^18^. This sampler consists of three subsections: a filter cassette that holds 9 discrete 25 mm filters with 0.22 μm polycarbonate (PC) membrane, an instrument housing, and a reagent housing. The filter-cassette design allows rapid removal and replacement of filters in the field by operators. The instrument housing contains the 26 valves, pump, pressure sensors, control circuitry, and inner fluid lines required for automating sample cleaning, collection, and preservation. The reagent housing is a free-flooded chamber that contains three separate IV bags that are preloaded with (a) RNase- and DNase-free water, (b) 5% HCl, and (c) RNAlater preservative. A fourth bag is initially empty and is used to capture waste reagents during the acquisition and preservation of the samples. The RNase- and DNase-free water is used for priming the system lines and filters when new filter cartridges are installed. The 5% HCl is used to sterilize and decontaminate the sample inlet and inner tubing before a sampling event. The RNAlater is loaded into filters after the sampling event to preserve both RNA and DNA. The waste bag is used to capture the preservative to ensure it does not enter the environment.

### Sample collection

**Fig. 1:**
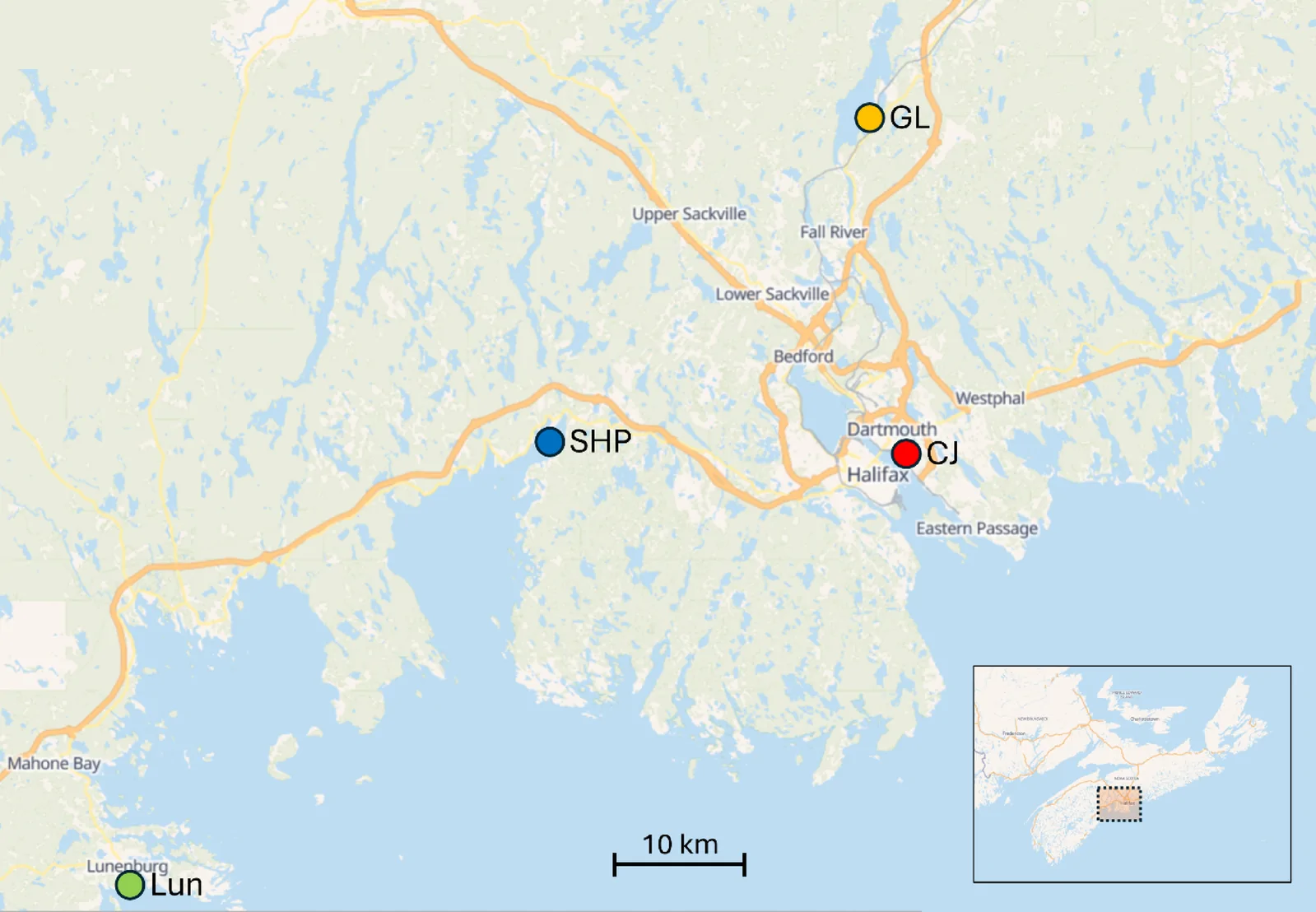
The four sampling locations in this study. CJ = Cove Jetty, GL = Grand Lake, Lun = Lunenburg, SHP = Sheeps Head Pond.

Water samples were collected at four locations in Nova Scotia (Fig. 1) in July 2023: Sheeps Head Pond (SHP: Tantallon, NS) on July 17, a jetty in Lunenburg Harbour (Lun) on July 19, Grand Lake (GL: Oakfield, NS) on July 21, and a jetty at the Centre for Ocean Venture and Entrepreneurship in Halifax Harbour (CJ: Dartmouth, NS) on July 22–24 (Table 1, Table S1). Sampler deployment was carried out in a similar manner to Hendricks et al.,^18^, with all handling of filters done using sterile technique with gloves. Prior to deployment, field equipment was cleaned and filters loaded into the filter cassette holder, which connects to the fluid manifold and syringe pump in the sampler. Samples were collected from SHP, Lun, and GL at approximately 45-minute intervals between 9:30 AM and 4 PM, with minor variation between sites. Sampling at CJ consisted of nine automated sample collection and preservation runs performed at four-hour intervals between 5:27 PM on July 22 and 12:02 AM on July 24.

**Table 1:** Summary of samples collected at each site, sampler, and sequencing marker. Int = interval in minutes or hours, Vol = sampled volume in mL. DOT and Niskin samples were relatively low in volume filtered, likely due to the high particulate content of the samples, indicating manual and automatic methods rapidly loaded the filter membrane.

Site	Sampler	Number of samples				Int	Vol
		16S	18S	12S	MG		
SHP	DOT	7	7	2	0	45 m	39.4
SHP	Niskin	7	7	0	0	45 m	42
Lun	DOT	9	9	2	0	45 m	99.1
Lun	Niskin	9	9	0	0	45 m	28
GL	DOT	9	9	2	0	45 m	40.7
GL	Niskin	9	9	0	0	45 m	52
CJ	DOT	9	9	2	9	4 h	108.1

After retrieval, preserved filters were removed from the cassette, LUER caps were installed on the inlet/outlet ports of each filter, then samples were bagged and stored frozen at −20 C. The sampler was powered by a DOT battery pack in all four locations and connected via a communication cable to a shore-based laptop for monitoring; however, such a connection is not required for autonomous sampling. The sampler was deployed into the water by rope in three of the four locations: in SHP, to a depth of 1 m from the back of a small inflatable vessel; in GL, to a depth of 1.5 m from a private dock; and in Lun, to a depth of 1.5 m from a commercial dock. In SHP, GL, and Lun, a Niskin bottle was lowered by rope into position next to the eDNA sampler and filled with sample water from the same location. A manual eDNA sample was then collected via a small peristaltic pump into an additional filter, with preservation post filtering. For the CJ samples, the DOT sampler was deployed via rope to a depth of 1.5 m using drop boards attached to the dock near the sewage outflow pipe.

### DNA/RNA extraction and sequencing

eDNA was extracted from frozen filters using the DNeasy Plant Mini Kit (Qiagen, Germany) with a modified lysis procedure, including a five-minute incubation at room temperature with 50 µL of 5 mg/mL lysozyme (MP Biomedicals, USA) followed by 1 h at 52 °C in 400µL Buffer AP1 supplemented with 45µL of 20 mg/mL proteinase K (Fisher BioReagents, United Kingdom). Extraction then proceeded following the manufacturer’s instructions, and eDNA was eluted in 50µL of Buffer AE. Two filters from each sample set were processed for eDNA and eRNA using the AllPrep^®^ Micro Kit (Qiagen, Germany). Cells were lysed via a 5 min incubation at room temperature with 50µL of 5 mg/mL lysozyme (MP Biomedicals, USA) followed by 15 min at 52 °C in 600µL Buffer RLT+ supplemented with 45µL of 20 mg/mL proteinase K (Fisher BioReagents, United Kingdom) and 12µL of 2 M dithiothreitol (Invitrogen, USA). An on-column DNase digestion was performed using the RNase-free DNase set (Qiagen, Germany). Cleaned, purified eRNA was eluted in 20µL of RNase-free water; eDNA was eluted in 50µL of Buffer EB. Nucleic acid concentrations and purity were assessed via a NanoDrop2000c (Thermo Scientific, USA). 6µL eRNA were treated with ezDNase and reverse-transcribed (RT) to cDNA using Superscript IV VILO (Invitrogen, USA) according to the manufacturer’s instructions; no-RT reactions were performed alongside to confirm the absence of any contaminating eDNA.

An amplicon sequencing strategy was designed to assess ecosystem biodiversity through detection of prokaryotic and eukaryotic sequences. For all eDNA and cDNA samples (RT and no-RT), the metabarcoding assays targeted regions of the prokaryotic and eukaryotic rRNA genes, 16S (V4-V5 regions^25^), and 18S (V4^26^), respectively. Additionally, for two DOT samples from each location, specific regions of eukaryotic mitochondrial (mt) genomes were targeted using the 12S MiFishU primers^27,28^. eDNA and cDNA for amplicon sequencing of 16S rRNA V4-V5 and 18S rRNA V4 regions were sent directly to the Integrated Microbiome Resource (IMR) facility at Dalhousie University and processed as described by Comeau et al.,^26^. PCR reactions targeting the 12S gene were performed in 20µL reactions using HotStar Taq (Qiagen, Germany), 250nM forward and reverse primers (IDT, USA), 200µM dNTPs (Invitrogen, USA), and 2µL eDNA template. 12 S reactions used 4 µg bovine serum albumin (NEB, USA) with touchdown cycling parameters of 15 min at 95 °C, 13 cycles of [30s 94 °C, 30 s 69.5 °C–1.5 °C/cycle, 90 s 72 °C], 30 cycles of [30s 94 °C, 30 s 50 °C, 45 s 72 °C], and 10 min at 72 °C. PCR products were purified with the Monarch spin PCR & DNA cleanup kit (NEB, USA) and submitted to the IMR for Illumina MiSeq sequencing (2 × 300 bp paired end).

Shotgun metagenomic sequencing was performed by the IMR for all 9 CJ samples. Libraries were prepared using the Illumina Nextera Flex kit as in Comeau and Filloramo^29^ and sequenced at 2X depth on the Illumina NextSeq2000 (2 × 150 bp paired end).

### Data analysis

All amplicons derived from environmental DNA and RNA were processed in the QIIME2 software package v2024.5.0^30^. All amplicons were trimmed and denoised using DADA2^31^. Taxonomic classification of 16S and 18S sequences was performed using the Naïve Bayes classifier with v138.1 of the SILVA database^32^. Only taxonomic assignments with a confidence score greater than 0.7 at a given rank were kept. For 12S, taxonomic assignment was performed using BLASTN against the MIDORI2 reference databases vGB266^33^ with all unique haplotype sequences included.

In the initial analysis, samples were rarefied to 1000 sequences each to maximize the number of retained samples. The Shannon diversity of all Niskin and DOT samples was computed and compared using a paired-sample *t*-test and a significance threshold of 0.05. Aitchison distances, which correct for the compositional nature of sequence data, were calculated between all samples and used as the basis for principal coordinates analysis (PCoA), both with the full dataset and with each site considered individually. The statistical significance of PCoA clusters was assessed using PERMANOVA with 999 randomizations, and dispersion tested with ‘betadisper’, both from the ‘vegan’ R package^34^. Taxonomy-based analyses (barplots, UpSet plots, heatmaps, and time-series visualizations) were performed using an abundance threshold *T*. In any given analysis, a taxonomic group at the appropriate rank with abundance $\:\ge\:$ *T* in at least one sample was included. The choice of rank and *T* was analysis specific.

Each sampled location had two time points where both 16S and 18S DNA and RNA were sequenced. Although the small sample size precluded statistical analysis, we compared DNA and RNA samples in a way that accounted for the compositional nature of amplicon sequence data. We first computed the centered log ratio (CLR) for each taxon * t* in each sample * s* using the standard formula:

$$\:\mathrm{C}\mathrm{L}\mathrm{R}\left({x}_{t,s}\right)=\mathrm{l}\mathrm{o}\mathrm{g}\left(\frac{{x}_{t,s}}{{g}_{s}}\right)$$

where $\:{x}_{t,s}$ is the count of taxon * t* in sample * s*, and $\:{g}_{s}$ is the geometric mean of all taxa in * s*. A pseudocount of 1 was added to each taxon to avoid taking the log of zero.

We then computed the CLR difference $\:{D}_{t,\mathcal{l}}$ for each taxonomic group * t* and location $\:\mathcal{l}$ as follows:

$$\:{D}_{t,\mathcal{l}}=\frac{1}{{n}_{\mathcal{l}}}\sum\:_{s\in\:\mathcal{l}}\left(\mathrm{C}\mathrm{L}\mathrm{R}\left({x}_{t,s}^{\mathrm{R}\mathrm{N}\mathrm{A}}\right)-\mathrm{C}\mathrm{L}\mathrm{R}\left({x}_{t,s}^{\mathrm{D}\mathrm{N}\mathrm{A}}\right)\right)$$

In our case, $\:{n}_{\mathcal{l}}=2$ (two paired DNA–RNA samples per location). We performed this comparison only on taxa that had nonzero abundance across all four samples at a given location, and further required that a taxon be present with a minimum abundance of at least * T* in one of the four samples.

For the metagenomic data generated from the 9 CJ samples, quality assessment and statistical analysis of raw reads was performed using FastQC v.0.12.1^35^. Subsequently, reads were processed using FastP v0.24.0^36^ to remove adapters, duplicates, and trim low-quality bases, with reads shorter than 50 bp after trimming discarded. The resulting high-quality reads were used for all subsequent downstream analyses. Assemblies were generated separately for each of the nine samples using MEGAHIT v1.2.9^37^ with the ‘–meta-large’ preset (k-mer sizes 27–127 bp, step size 10). Assembled contigs shorter than 1000 bp were discarded. Assembly quality was evaluated using QUAST v5.2.0^38^ with the MetaQUAST extension, generating metrics including the number of contigs, total assembly length, largest contig, and N50.

Taxonomic classification of processed reads and contigs was performed with Kraken2 v2.1.2 using default parameters^39^. Functional profiling was carried out with HUMANn v3.8^40^, which assigned annotations for MetaCyc reactions, Gene Ontology (GO) terms, and KEGG Orthology (KO) identifiers^41^. These annotations were further processed using KEGG-decoder v1.3.0^42^ to assess the presence and completeness of metabolic pathways. Detection of antimicrobial-resistance genes (ARGs) was performed using the Resistance Gene Identifier (RGI) v6.0.5 from the Comprehensive Antibiotic Resistance Database (CARD) on contigs^43^. RGI Perfect and Strict matches were then compared against the NCBI clustered non-redundant protein database (2025/07/05 release) using BLASTP version 2.16.0 through the NCBI online portal (National Center for Biotechnology Information^44^ with default parameters.

## Results

Sequencing of 16S, 18S, and 12S DNA from all DOT samples yielded an average of 24,634 amplicons per sample (*n* = 76), versus 18,650 for the Niskin samples (*n* = 50) (Table 1, Table S1). AllPrep^®^ extractions yielded an average of 94,931 raw sequences per sample obtained from 16S to 18S cDNA sequencing of DOT samples, versus 74,984 for the Niskin samples. Metagenome sequencing of DOT COVE Jetty samples yielded an average of 16,680,966 reads across nine samples. Repeated rarefaction curves (Fig. S1) suggested that the sampled amounts were sufficient to recover most ASVs from all habitats.

**Fig. 2:**
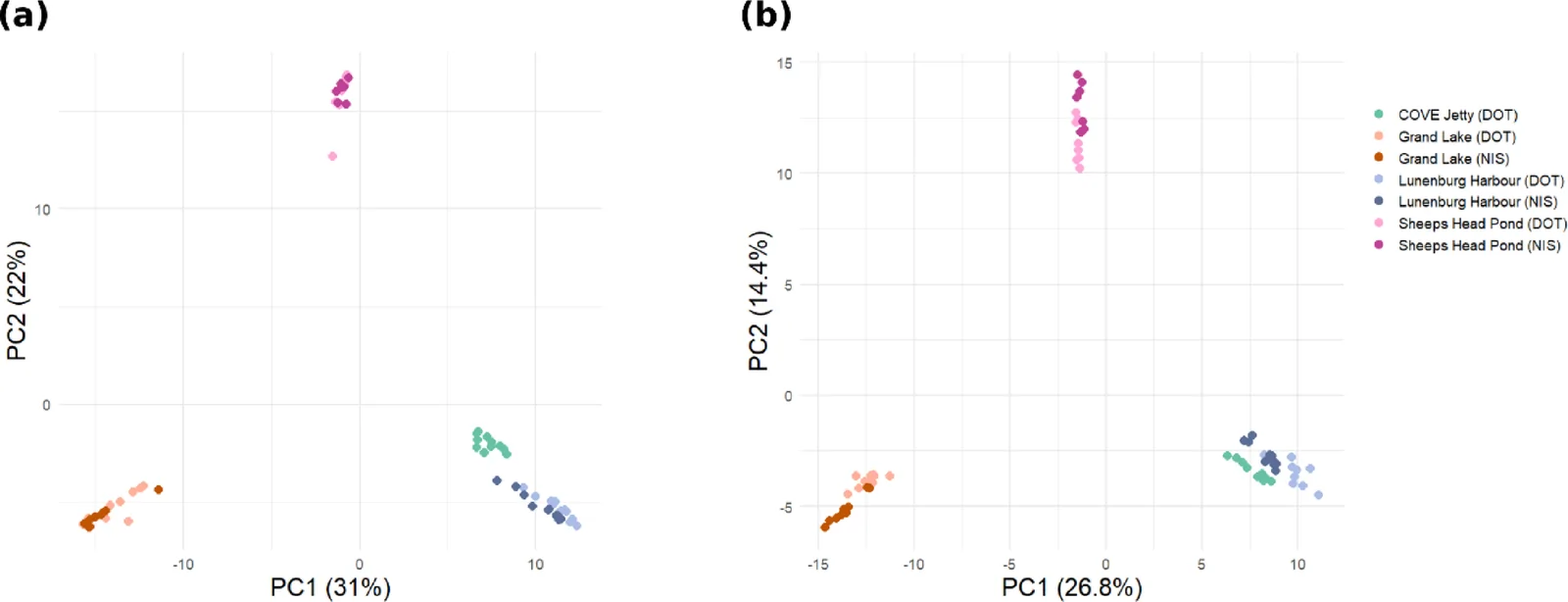
Principal coordinates analysis of DOT and Niskin samples. Ordinations of 16S (**a**) and 18S (**b**) samples are shown. Location colours are consistent with Fig. 1.

Principal coordinates analysis showed differentiation of sites (Fig. 2), with substantial overlap between DOT and Niskin samples. The two marine sites, Lun and CJ, were close to one another in the PCoA plots but still formed separate clusters. PERMANOVA tests by location were statistically significant (16S: *p* = 0.001, R^2^ = 0.642; 18S: *p* = 0.001, R^2^ = 0.53), with differences in dispersion between sites (*p* = 0.001 for both 16S and 18S). PCoA plots separated DNA from RNA sequences at most sites (Fig. S2), with PERMANOVA R^2^ values ranging from 0.111 (Lun) to 0.152 (SHP) for the 16S markers, and 0.09 (Lun) to 0.181 (CJ) for 18S. Sampler effects tended to be lower in 16S (SHP R^2^ = 0.082, GL = 0.075, Lun = 0.282) than 18S (SHP R^2^ = 0.426, GL = 0.166, Lun = 0.32). Patterns in ASV Shannon diversity differed by site (Fig. S3), with the DOT sampler showing higher diversity than Niskin in Lun (16S: *p* = 0.0005; 18S: *p* = 0.0009), but lower in GL (16S: *p* = 0.036; 18S: *p* = 0.001) and no difference in SHP (16S: *p* = 0.876; 18S: *p* = 0.806).

16S profiles (Fig. S4a) clustered perfectly by site rather than by sampler, suggesting consistent taxonomic recovery between sampling approaches. However, the 18S profiles (Fig. S4b) did not cleanly separate CJ from Lun, and there was stronger clustering by sampler, consistent with the PCoA plots in Fig. S2. 12S profiles, all based on DOT samples, also separated samples by location (Fig. S4c).

### Sheeps Head Pond

Sheeps Head Pond is located on a small island in Tantallon, Nova Scotia situated in the headwaters of St. Margarets Bay. The pond was originally an inlet of the ocean but was cut off by the construction of causeways in the mid-20th century. Although the pond remains connected to the ocean via culverts, the pond freezes during the middle of the winter. Residents have expressed concerns about sulfurous smells in spring and fall, suggesting the potential presence of sulfate-reducing bacteria.

Observed taxa largely overlapped between DOT DNA and RNA samples in SHP: 25 families from 16 S and 10 species from 12 S were always present (Fig. S5). Nine phyla were observed in all 18 S DNA and RNA sequence sets except sample SHP-1, in which only 179 18 S sequence reads passed quality-control filters. DOT and Niskin samples were not differentiated in the 16 S profiles, while there was complete separation between DOT and Niskin samples in the 18 S phylum data, with DOT time point 1 as an outlier. The overrepresentation of Ascomycota in the DOT samples likely contributes to the underrepresentation of other groups such as Arthropoda, Rotifera, and Platyhelminthes relative to the Niskin samples (Fig. S6).

**Fig. 3:**
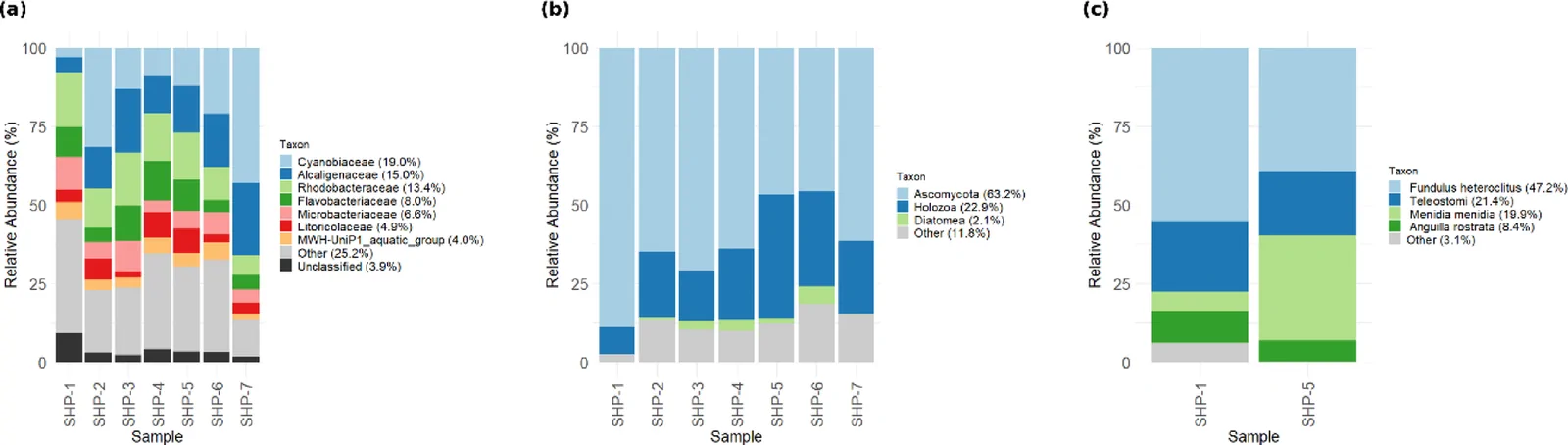
Distributions of taxa based on analysis of marker genes in Sheeps Head Pond. (**a**) 16 S profiles, family level, minimum abundance $\:\ge\:$ 5% in at least one sample. (**b**) 18 S profiles, phylum level, minimum abundance $\:\ge\:$ 5% in at least one sample. (**c**) 12 S profiles, species level, minimum abundance $\:\ge\:$ 1% in at least one sample.

The top 16 S families by relative abundance across DOT samples were Cyanobiaceae (19% average abundance), Alcaligenaceae (15%), and Rhodobacteraceae (13.4%) (Fig. 3a). Phylum Desulfobacterota^45^, which includes many SRBs, accounted for an average of 0.3% of all sequences (Fig. S7a). While most of the identified families contain sulfate-reducing bacteria, Bradymonadales, which accounted for > 75% of assigned sequences in this phylum, is not directly associated with hydrogen sulfide production^46^. The classes Sordariomycetes and Saccharomycetes accounted for 89.1% of all ascomycete sequences (Fig. S7b); both have been observed as contaminants in water lines and are unlikely to comprise significant biomass in a brackish pond^47,48^. Other common phyla included Holozoa, Ciliophora, and Diatomea, with animal phyla including Cnidaria and Gastrotricha in lower abundance (Fig. 3b). Profiling of the 12 S rRNA gene suggested the presence of fish associated with brackish water and diadromous migrators (Fig. 3c). The most abundant of these was the mummichog *Fundulus heteroclitus*, which had an average abundance of 47.2% across the two samples, followed by sequences classified only to the level Teleostomi (21.4%), a group that includes all bony fish and tetrapods. Other species observed include the Atlantic silverside *Menidia menidia* (19.9%), the American eel *Anguilla rostrata* and unclassified *Anguilla* (9.8% total), and Atlantic herring *Clupea harengus* (1.2%). CLR-based comparisons of DNA vs. RNA samples showed the eukaryotic phyla Prymnesiophyceae, Cercozoa, and Diatomea as most overrepresented in RNA sequences, while the bacterial families Pirellulaceae and Acholeplasmataceae, and phylum Cnidaria were underrepresented (Fig. S8).

### Grand Lake

Shubenacadie Grand Lake (Mi’kmaw: Kji-qospem) is a freshwater lake in Nova Scotia, approximately 35 km north of Halifax. A popular recreation area, Grand Lake contains fish including the invasive chain pickerel *Esox niger* and the smallmouth bass *Micropterus dolomieu*. Cyanobacterial blooms are a significant concern, and a recent two-year sampling program identified the benthic cyanobacterium *Microcoleus* at several locations, suggesting that mats may have been transported to the lake shore after a heavy rainfall event^49^.

Taxonomic profiles from the 16 S marker showed a striking pattern, with 21 taxa observed above the minimum threshold in the two RNA profiles but missing from all DNA profiles (Fig. S9), with an additional 16 families uniquely found in one of the two RNA profiles. Sixteen families were present in all DNA and RNA samples, or missing only from sample GL-6, which had only 188 sequences that passed quality control. Most taxa inferred from the 18 S and 12 S markers were present in all samples. Bacterial profiles for DOT and Niskin samples were similar, while the DOT samples contained more Ascomycota and fewer samples with Rotifera, Centrohelida, and Diatomea relative to Niskin samples (Fig. S10).

**Fig. 4:**
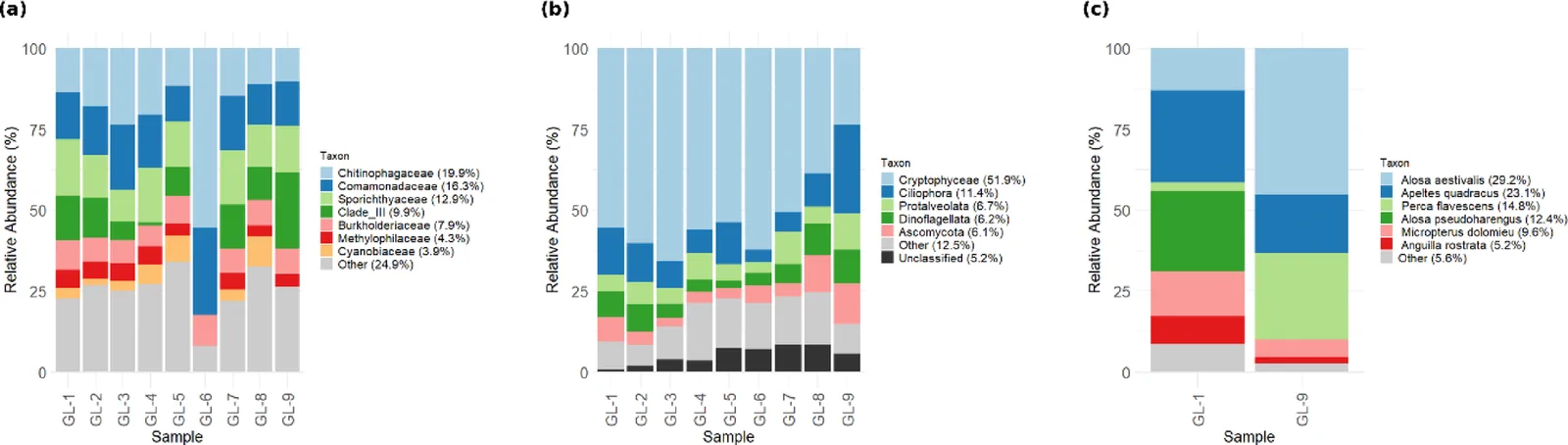
Distributions of taxa based on analysis of marker genes in Grand Lake. (**a**) 16 S profiles, family level, minimum abundance $\:\ge\:$ 5% in at least one sample. (**b**) 18 S profiles, phylum level, minimum abundance $\:\ge\:$ 5% in at least one sample. (**c**) 12 S profiles, species level, minimum abundance $\:\ge\:$ 1% in at least one sample.

16 S profiles were dominated by the families Chitinophagaceae, Comamonadaceae, and Sporicthyaceae, all common constituents of freshwater lakes^50,51^ (Fig. 4a). Cyanobiaceae, the lone cyanobacterial family, had an average relative abundance of 3.9%; this family is frequently observed in the water column and is not implicated in harmful algal blooms^52,53^ (Fig. S10). The 18 S profiles were dominated by the cryptomonad algae Cryptophyceae (51.9%) and Ciliophora (Fig. 4b). The 12 S profiles showed a range of expected freshwater species (Fig. 4c), including native species of shad, stickleback, alewife, American eel, and brown bullhead, the invasive smallmouth bass, and the pike family Esocidae, which includes the chain pickerel and other species. Many taxa had lower abundance in RNA than in DNA sequences, including the bacterial families Sporicthyaceae and Ilumatobacteraceae, while Bdellovibrionaceae and Rhodobacteraceae were more abundant (Fig. S11a). Fewer differences were observed in the 18 S comparisons, with phyla Dinoflagellata, Protalveolata, Ascomycota, and Chytridomycota less abundant in RNA sequences (Fig. S11b).

### Lunenburg Harbour

Lunenburg, Nova Scotia, (Mi’kmaw: E’se’katik, “place of clams”) is a town with 2400 inhabitants on the South Shore of Nova Scotia. The town has recently developed an extensive program of wastewater-treatment upgrades and outfall relocations to address prior issues of fecal contamination (Town of Lunenburg^54^). Shipping and other industries have led to significant impacts from invasive species including the European green crab *Carcinus maenas*, the European sea squirt *Ascidiella aspersa* and compound sea squirt *Diplosoma listerianum*^55^, and invasive red algae *Dasysiphonia (Heterosiphonia) japonica*^56^.

Twenty-four prokaryotic families were observed across all DNA and RNA samples, with eleven taxa found exclusively in one or both RNA profiles (Fig. S12a). Seventeen phyla inferred from 18 S sequences were present in all DNA and RNA samples, with an additional six restricted to one or both RNA profiles (Fig. S12b). Seven of eleven species inferred from 12 S were present in both profiled samples (Fig. S12c). Heatmaps confirmed the presence of most taxa across most or all DOT and Niskin samples at a lower abundance threshold (Fig. S13a). The 18 S phyla followed a similar pattern, with all but five phyla identified in 14/18 samples (Fig. S13b). The eukaryotic phyla Ascomycota, Tunicata, and Picozoa were overrepresented in the DOT samples relative to Niskin.

**Fig. 5:**
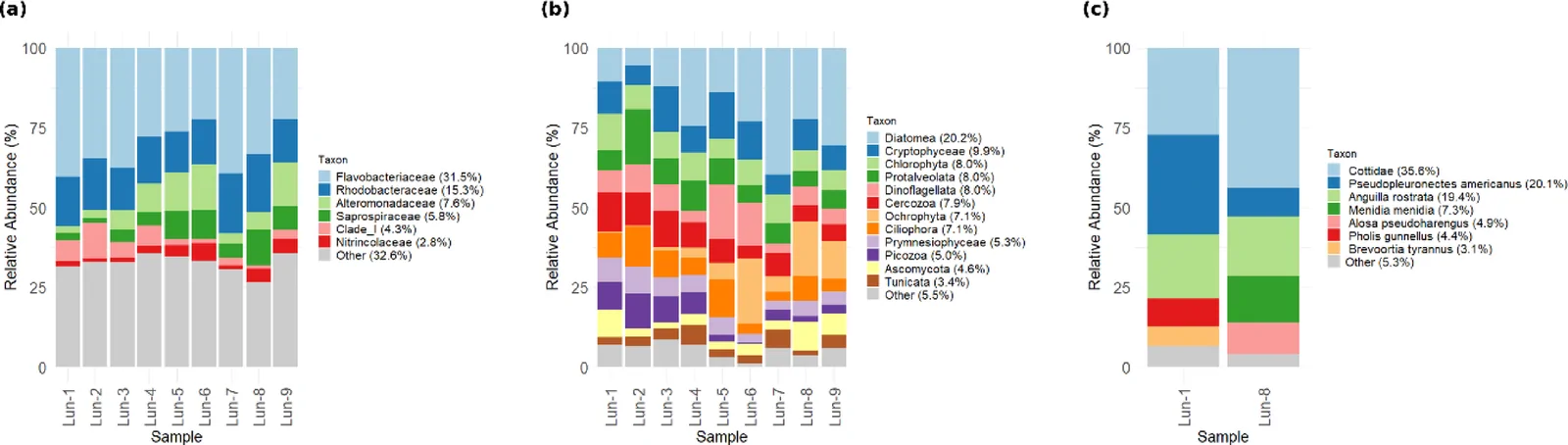
Distributions of taxa based on analysis of marker genes in Lunenburg Harbour. (**a**) 16 S profiles, family level, minimum abundance $\:\ge\:$ 5% in at least one sample. (**b**) 18 S profiles, phylum level, minimum abundance $\:\ge\:$ 5% in at least one sample. (**c**) 12 S profiles, species level, minimum abundance $\:\ge\:$ 1% in at least one sample.

**Fig. 6:**
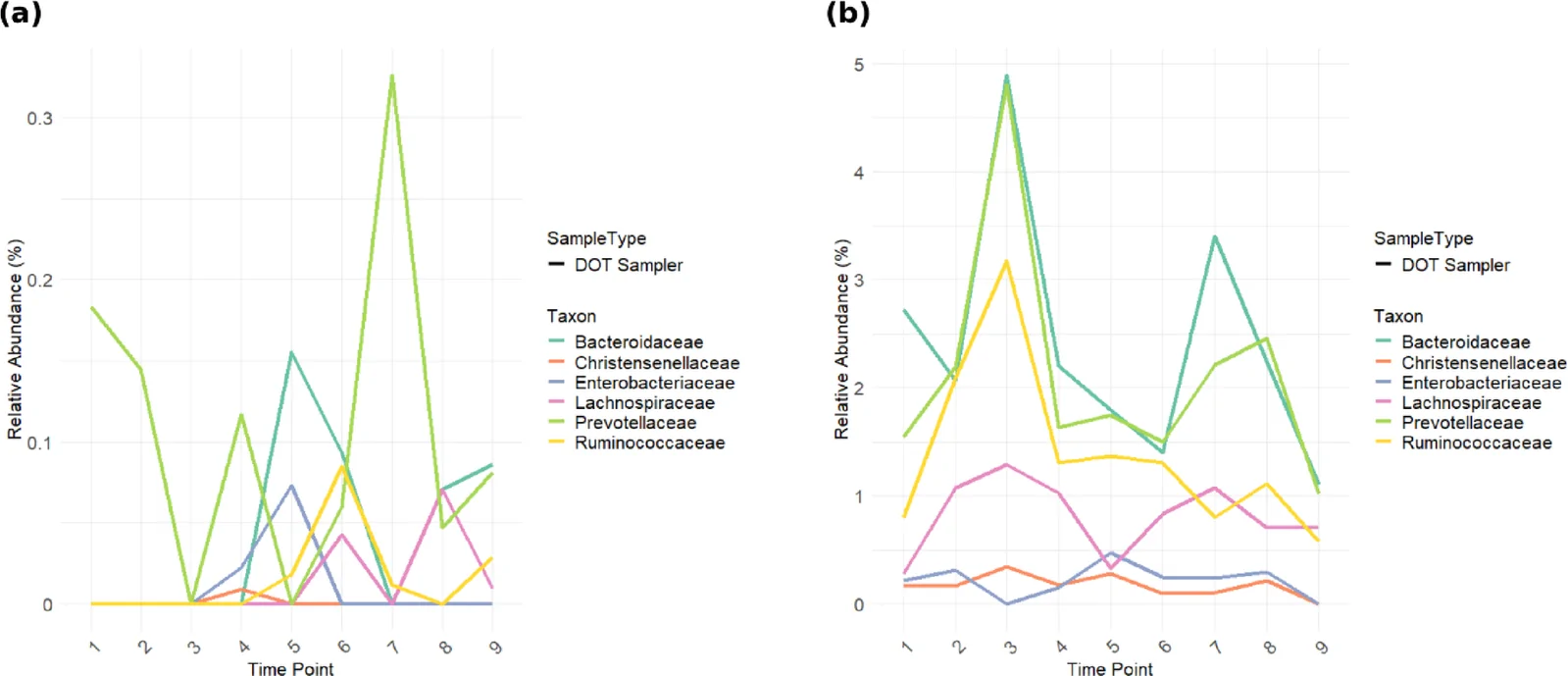
Relative abundance of six bacterial families across nine time points in samples collected from Lunenburg Harbour (**a**) and COVE Jetty (**b**). A seventh family, Enterococcaceae, was absent from all collected samples.

The most common bacterial families included Flavobacteriaceae, Rhodobacteraceae, and Alteromonadaceae (Fig. 5a). We further examined the relative abundance of seven bacterial families often associated with the guts of humans and other animals (Fig. 6a): the maximum abundance of any of these groups was 3.3% (Prevotellaceae at time-point 7), with no other observation > 0.2%. Enterococcaceae, identified in previous studies as the key fecal contaminant in Lunenburg Harbour, was not detected at any time point. 18 S phyla included diatoms, cryptophytes, and chlorophyte algae, as well as marine-specific lineages including haptophyte algae and Picozoa (Fig. 5b). Tunicates were detected in all samples. 12 S profiling showed a range of known marine and brackish taxa, including sculpins (Cottidae), the winter flounder *Pseudopleuronectes americanus*, and the American eel *Anguilla rostrata* (Fig. 5c). Differences in 16 S DNA and RNA abundance were less pronounced than in Grand Lake, with the common taxon Saprospiraceae overrepresented in RNA (Fig. S14a). Phylum Prymnesiophyceae was overrepresented in 18 S RNA sequences, whereas Protalveolata and Ascomycota were again underrepresented (Fig. S14b).

### COVE Jetty, Halifax Harbour

Halifax, Nova Scotia (Mi’kmaw: Kjipuktuk, “great harbour”), is the largest city on the east coast of Canada, with approximately 500,000 inhabitants. The Bedford Basin has long been studied by the Department of Fisheries and Oceans and by Canadian and international researchers^57,58^, with sequencing of 16 S and 18 S genes from weekly samples conducted since 2014^21,59^. Wastewater treatment upgrades in the late 2000s resulted in improved water quality and lower microbial loads, but in times of high rainfall, sewage is still released into the harbour. On July 21–22, 2023, Nova Scotia experienced a massive rainfall event with up to 250 mm falling in parts of the Halifax Regional Municipality in a 24-hour period, which led to catastrophic flooding (CTV News Atlantic^60^). On July 22, we deployed a DOT sampler near a sewage outflow to assess potential increases in human-associated bacteria.

Thirty-one prokaryotic families were found in all DNA and RNA samples (Fig. S15). Fifteen 18 S phyla were ubiquitous. Similarly, nine out of eleven species identified using 12 S were found in both samples. Two families typically associated with the human microbiome, Ruminococcaceae and Lachnospiraceae, were present with abundance $\:\ge\:$ 1% in six and four profiles, respectively, although both were absent from the RNA profiles (Fig. S16). The animal phyla Tunicata, Mollusca, and Holozoa were seen consistently across all or nearly all samples (Fig. S16b).

**Fig. 7:**
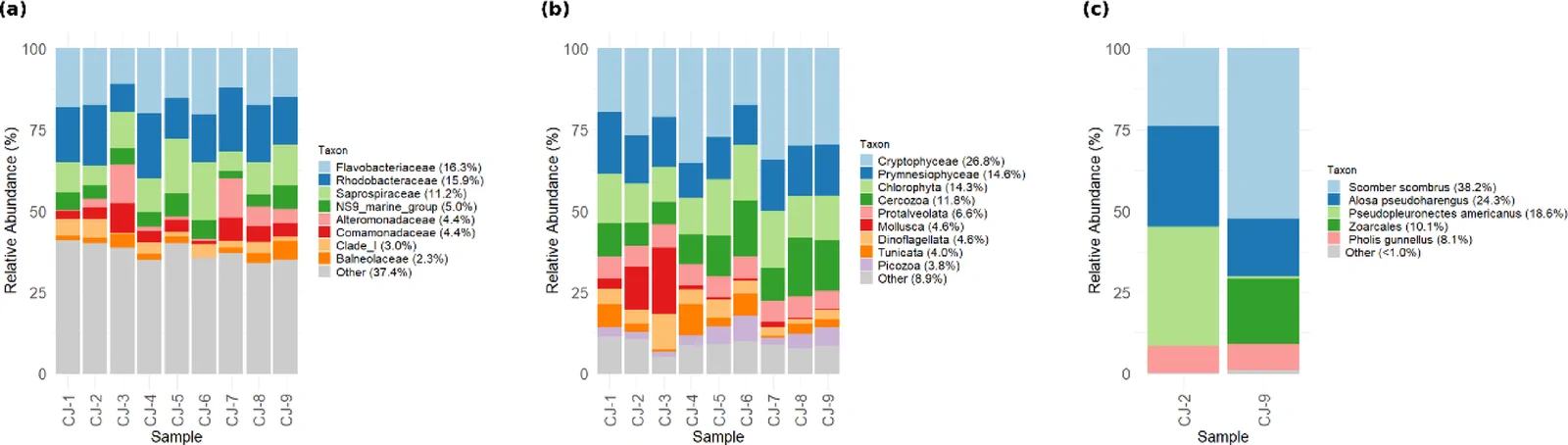
Distributions of taxa based on analysis of marker genes in COVE Jetty. (**a**) 16 S profiles, family level, minimum abundance $\:\ge\:$ 5% in at least one sample. (**b**) 18 S profiles, phylum level, minimum abundance $\:\ge\:$ 5% in at least one sample. (**c**) 12 S profiles, species level, minimum abundance $\:\ge\:$ 1% in at least one sample.

The dominant bacterial families in CJ were similar to those found in Lunenburg Harbour (Fig. 7a). However, three other families were found in at least one CJ sample with a relative abundance $\:\ge\:$ 5%: the NS9 marine group, Comamonadaceae, and Balneolaceae. Four of the seven bacterial phyla often associated with the human gut were present at a relative abundance > 1% in at least one CJ sample. Bacteroidaceae was the eighth most-abundant bacterial family overall (Fig. 6b). Five families peaked in relative abundance at time-point 3, with Bacteroidaceae and Prevotellaceae at an abundance of nearly 5%, Ruminococcaceae above 3%, and Lachnospiraceae above 1%. 18 S phylum-level profiles were similar to those found in Lun, albeit with different average relative abundance values: for example, Cryptophyceae (CJ = 26.8%, Lun = 9.9%), Prymnesiophyceae (CJ = 14.6%, Lun = 5.3%), and Chlorophyta (CJ = 14.3%, Lun = 8%) (Fig. 7b). Fewer species were present with relative abundance $\:\ge\:$ 1% in the 12 S profiles: the Atlantic mackerel *Scomber scombrus* (CJ: 38.2%, Lun: 3.2%), the alewife *Alosa pseudoharengus* (CJ: 24.3%, Lun: 4.9%), the winter flounder *Pseudopleuronectes americanus* (CJ: 18.6%, Lun: 20.1%), and the rock gunnel *Pholis gunnellus* (CJ: 8,1%, Lun: 4.4%). Additionally, 0.1% of CJ 12 S sequences were assigned to the order Zoarcales, which may also correspond to gunnels. Very few taxa showed absolute CLR differences greater than 0.5, with Moraxellaceae overrepresented and Clade I (order SAR11) underrepresented (Fig. S17a). No differences above the CLR threshold were observed in the 18 S data (Fig. S17b).

The nine metagenomic assemblies had N50s between 4749 and 8350, with the longest contigs ranging in size between 129,130 and 415,927 nt and assigned to genera *Planktomarina*, *Pseudomonas*, *Flavobacterium*, and *Alteromonas*. All but *Pseudomonas* were among the five most-abundant families from the 16 S profiles. Relative-abundance estimates differed dramatically between 16 S and metagenomic analysis (Fig. S18), with Pelagibacteraceae absent from the 16 S profiles but most abundant in the metagenomic reads and contigs. Conversely, several families from Proteobacteria and Bacteriodota and OM190, the lone family from Planctomycetota, were found in the 16 S but not the metagenomic samples.

**Fig. 8:**
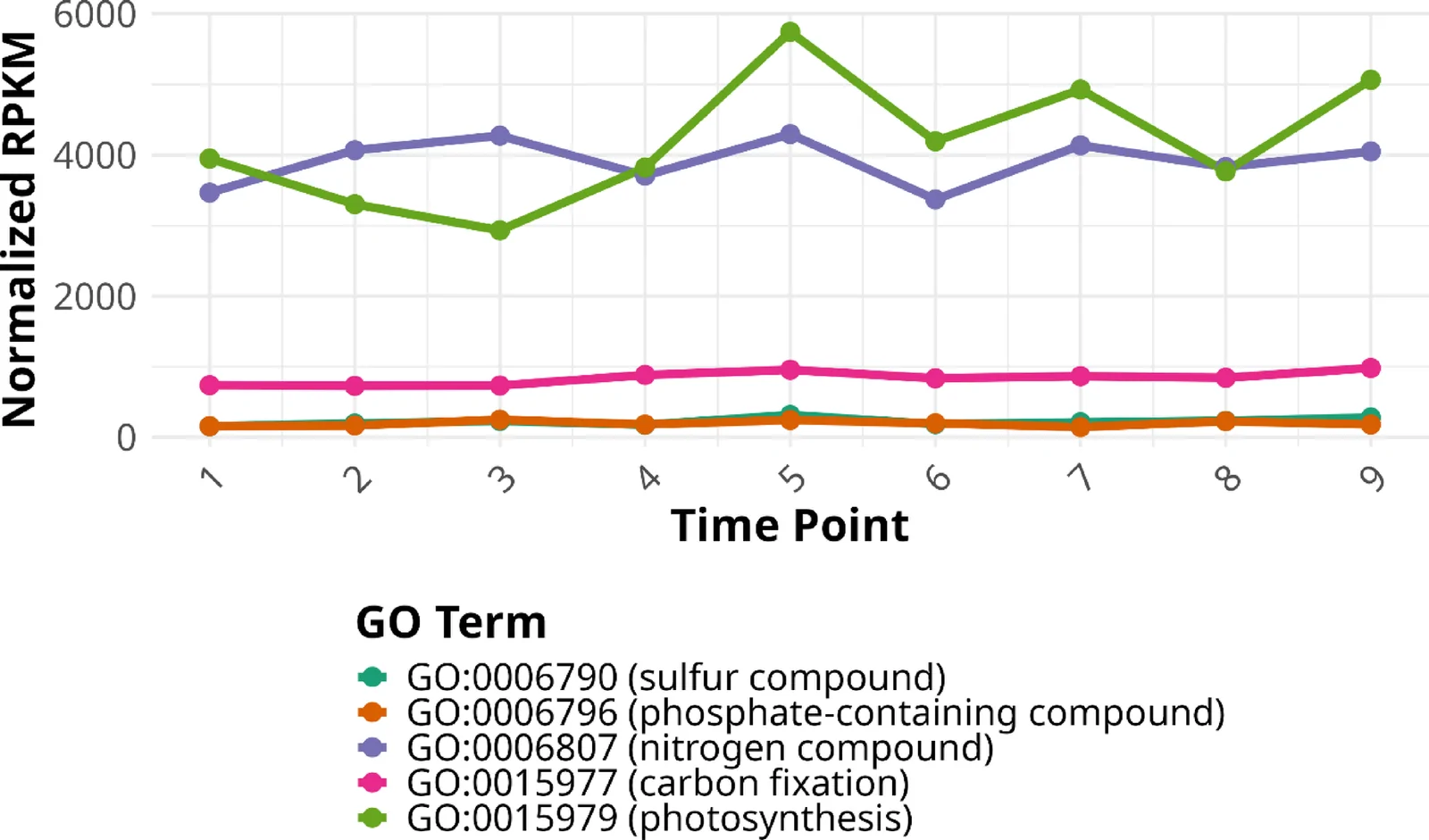
Normalized read counts of genes associated with key biogeochemical cycling pathways and photosynthesis, inferred using HUMAnN3.

Functional profiling of key GO pathways yielded normalized estimates of relative pathway abundance. Key nutrient-cycling pathways including photosynthesis, sulfur, phosphate, nitrogen, and carbon metabolism (Fig. 8), none of which showed an increasing, decreasing, or cyclic trend. We also used RGI with the CARD database to assess the presence of putative antimicrobial-resistance genes (ARGs) from the assembled contigs. A maximum of 15 Perfect and Strict ARGs were predicted in any sample, with glycopeptide and disinfecting agents comprising 78.8% of all predicted genes (Tables S3-S6). However, percent identities for most resistance classes were generally low (< 50%), apart from five genes predicted to confer resistance to macrolides and streptogramins (100% identity to reference sequence), which mapped to the family Enterobacteriaceae, and four genes labeled “fluoroquinolone; diaminopyrimidine; phenicol” (86.7% identity) predicted to be present in Vibrionaceae. The apparent rarity and poor conservation of ARGs is consistent with the taxonomic profile of the samples, where most reference CARD genes are from pathogenic organisms (e.g., Enterobacteriaceae), while the CJ samples are dominated by classes such as Alphaproteobacteria that are poorly represented or absent from validated reference databases of ARGs.

## Discussion

We successfully used the DOT eDNA sampler to profile prokaryotic and eukaryotic biodiversity across a variety of aquatic habitats, and to address specific ecological questions at each site. There was no evidence that the samples with lowest retained read counts were outliers in any diversity analysis, which demonstrates that even small volumes of water can yield sufficient DNA and RNA concentrations for metabarcoding and metagenomics. We observed a high degree of concordance in species assemblages between the DOT and Niskin samples across habitats and environments (Fig. S2), further demonstrating the utility of automated sampling and the DOT sampler itself for reconstructing patterns of biodiversity through space and time. This high concordance was observed even in the presence of ascomycete fungal contamination present at the SHP location fungi in the 18 S profiles. Overall, differences in diversity estimates between the two sampling approaches were not systematic (Fig. S3), further demonstrating the effectiveness of automated eDNA sampling to enhance eDNA-based biomonitoring.

### Contrasting eDNA and eRNA

We obtained high eDNA and cDNA recovery from the AllPrep^®^ kits (average sequencing depth = 83,541 reads/sample). Taxonomic profiles inferred from eRNA were often more similar to each other than they were to the matched eDNA samples. eRNA in our study yielded higher species richness, particularly at the microbial level, suggesting it better reflects species composition of active microorganisms at the time of sampling. eRNA has demonstrated higher sensitivity of fish detection^61^; while this result might seem counterintuitive given the instability of RNA, several factors may increase its persistence, including differences in particle association and higher copy numbers of ribosomal RNA versus DNA in metabolically active cells^62^. Different marker-gene copy numbers and differential regulation will also contribute to observed differences across taxa, as will the choice of minimum abundance threshold used for analysis of taxa. Sample preservation using the DOT sampler will also enable metatranscriptomic analysis to assess the expressed functional profile of the microbiome^16,63^.

### Location-specific insights

PCoA and clustering analyses strongly supported the species turnover between freshwater, brackish, and marine sites. Using multi-marker analysis, we were able to identify both native and invasive species, as well as environmentally relevant microorganisms. No single taxon exceeded ≥ 5% eDNA reads at all four sites although several microbial taxa, along with American eel (*Anguilla rostrata*: SHP, GL, Lun) and alewife (*Alosa pseudoharengus*: GL, CJ, Lun) exceeded this threshold at three sites. Notably, both fish species were found in both marine and freshwater environments, demonstrating habitat breadth and connectivity. Many prokaryotic families and eukaryotic phyla were present across all four sites at a lower minimum relative abundance of 0.1%. Detection of specific animal taxa in a range of distinct habitats demonstrates the potential role of the DOT sampler in assessing the ecological connectivity between sites, a key component of ecological modeling and risk assessment.

#### Sheeps Head Pond

The brackish Sheeps Head Pond contained a mix of taxa associated with marine, fresh, and brackish waters. These include Cyanobiaceae, Rhodobacteraceae, and Litoricolaceae, and small but detectable levels of Desulfobacterota that may be responsible for the reported smell of sulfur. The probable contaminants from the ascomycete classes Sordariomycetes and Saccharomycetes decreased the relative abundance of other taxa inferred from the 18 S profiles, but most phyla were nonetheless recovered at all or nearly all sites, including Holozoa, Rotifera, and Cnidaria, with Cnidaria substantially underrepresented in RNA sequencing, suggesting potential mortality of marine jellyfish before or after arrival in the brackish environment. 12 S profiling detected fish species associated with brackish conditions, including the coastal-associated mummichog, Atlantic silverside, and American eel.

#### Grand Lake

As expected, bacterial taxa observed in Grand Lake such as Chitinophagaceae, Comamonadaceae, and Sporicthyaceae were primarily freshwater associated. The key cyanobacterial HAB taxon *Microcoleus*, previously identified in the water column by Johnston et al.,^49^ was not detected in any of our Grand Lake samples, although we did detect the family Cyanobiaceae at low relative abundance. Our findings suggest a low immediate risk of HABs, although a more-comprehensive survey of inlets and outlets of the lake would be necessary to precisely assess baseline risk. DOT samples also recovered 18 S DNA from the freshwater-associated Cryptophyceae, Ciliophora, and Protalveolata in the freshwater lake, with animal taxa including Holozoa, Porifera, Mollusca, and Rotifera. 12 S profiling identified yellow perch, smallmouth bass, and pike. The large number of taxa observed only in eRNA samples suggests a rare but metabolically active biosphere, which may include instances of conditionally rare taxa that are increasing in abundance. Future work with the DOT sampler could involve sampling over several days, and comparing observed eRNA abundance with increases and decreases in the corresponding microbial populations.

#### Lunenburg Harbour and COVE Jetty

Most taxa in Lunenburg Harbour and at the COVE Jetty were marine associated, including the bacterial families Flavobacteriaceae, Rhodobacteraceae, and Alteromonadaceae, and microbial eukaryotes from the phyla Diatomea, Cryptophyceae, Chlorophyta, Cercozoa, and Protalveolata. Additionally, the COVE Jetty had signatures of potential runoff and wastewater-associated bacteria such as Comamonadaceae and the probable human-associated families shown in Fig. 6. The seven human microbiome-associated families examined were approximately ten times higher in abundance by sequence depth in the COVE Jetty samples than in Lunenburg Harbour. This pattern may reflect improved wastewater treatment in Lunenburg and increased sewage discharge into the Halifax harbour following a major rainfall event, but thorough tests with baselines will be necessary to demonstrate this more conclusively. Additionally, animal phyla including Mollusca and potentially invasive Tunicata were identified in both harbours, with Arthropoda and Annelida additionally present in Lunenburg Harbour. 12 S profiling identified fish species at both sites, including the rock gunnel *Pholis gunnellus* and winter flounder *Pseudopleuronectes americanus*.

Metagenomic analysis of COVE Jetty samples identified key nutrient-cycling pathways at similar relative abundances across the nine time points sampled, despite changes in the relative abundance of probable human-associated taxa. Taxonomic profiling of metagenomic versus 16 S sequence data was highly inconsistent (Fig. S17), with many abundant taxa inferred from 16 S showing lower abundance or complete absence from the metagenomic predictions. This inconsistency is well documented^64–67^, (Cordier et al., 2021), and a combined approach can provide complementary information on microbial diversity. Although the recovered taxonomic and functional profiles were relatively stable over the sampling period, automated deployments that collect samples before, during, and after a disturbance can provide information about community stability, resilience, and functional redundancy. AMR analysis (Tables S4-S7) identified few plausible genes but illustrates the ability to profile specific genes of interest from preserved samples.

### Further development

The filter-cassette design of the DOT sampler allows for rapid swapping out of filters in the field, with the additional benefit of minimizing re-engineering to accommodate a variety of different filter types. The sample volumes collected in this study were sufficient to carry out detailed community profiling; however, detection of very rare organisms using techniques such as qPCR will likely require larger sample volumes. To address this with the DOT system, Luy et al.,^68^ demonstrated the use of 45 mm diameter filters that can capture more than 5 L per sampling event, although turbidity can fundamentally limit flow across any filter. Automated cleaning, inlet flushing, and sterilization of the fluid lines help ensure that samples captured on the filter membrane/material are representative of the water sample during the time window of capture. Based on the SHP results shown here, an updated cleaning protocol based on 5% HCl has been implemented on the samplers and validated in other studies, eliminating cross-contamination^9^. Successful sample preservation also allows for other profiling techniques such as transcriptomics, targeted profiling using qPCR, or sequencing while aboard research vessels or in remote locations.

Our study further supports the use of autonomous sampling technologies for multiple genetic analyses across different environments. The COVE Jetty provides a particularly compelling case study: during the extreme flooding event, manual sampling would have posed significant safety risks, while maintaining regular, around-the-clock collections would have been logistically unfeasible. With the DOT sampler, we were able to monitor the site through repeated, unattended sampling, capturing the presence of human-associated bacteria following the discharge event. Our work provides a key validation of automated eDNA technology, and pilot data that shed light on important ecological questions in multiple aquatic habitats. In the Nova Scotian context specifically, automated sampling can support monitoring activities including the impact of major adverse weather events such as cyclones^69^; continuous assessment of species diversity in nearshore and offshore marine protected areas, and salmon rivers^22,23,70^; detection of aquatic invasive species in harbours and lakes^71^; and monitoring for species associated with harmful algal blooms^49^. Nova Scotia is also investing heavily in offshore wind, a sector that is increasingly using eDNA as a tool for environmental monitoring. Integration of the DOT sampler with autonomous surface and underwater vessels will allow for year-round monitoring at a greatly reduced cost relative to crewed deployments.

## Supplementary Information

Below is the link to the electronic supplementary material.

Supplementary Material 1

## Data Availability

Sequence data are available at the NCBI Sequence Read Archive under the Bioproject ID PRJNA1417789.

## Abbreviations

ASV: Amplicon sequence variant
ARG: Antimicrobial resistance genes
CARD: Comprehensive Antibiotic Resistance Database
eDNA/eRNA: Environmental DNA/RNA
16S/18S/12S: 16S/18S/12S ribosomal RNA gene
SHP: Sheeps Head Pond, Nova Scotia
GL: Shubenacadie Grand Lake, Nova Scotia
Lun: Lunenburg Harbour, Nova Scotia
CJ: COVE Jetty, Halifax Harbour, Nova Scotia
CLR: Centered log‐ratio transformation
DOT: Dartmouth Ocean Technologies, Inc.
