# Supplementary material for: Automated eDNA and eRNA profiling for biodiversity monitoring in marine and freshwater ecosystems
Source: Sci Rep. 2026 Jun 25;16:29184. doi: 10.1038/s41598-026-58421-1 (PMC13590487; doi:10.1038/s41598-026-58421-1)
Supplement: Supplementary file 1 [file 41598_2026_58421_MOESM1_ESM.pdf]

Received: 21 January 2026 | Revised: 5 May 2026 | Accepted: 15 June 2026

DOI: https://doi.org/10.1038/s41598-026-58421-1

Supplementary Information for "Automated eDNA and eRNA Profiling for Biodiversity Monitoring in Marine and Freshwater Ecosystems"

 © The Authors

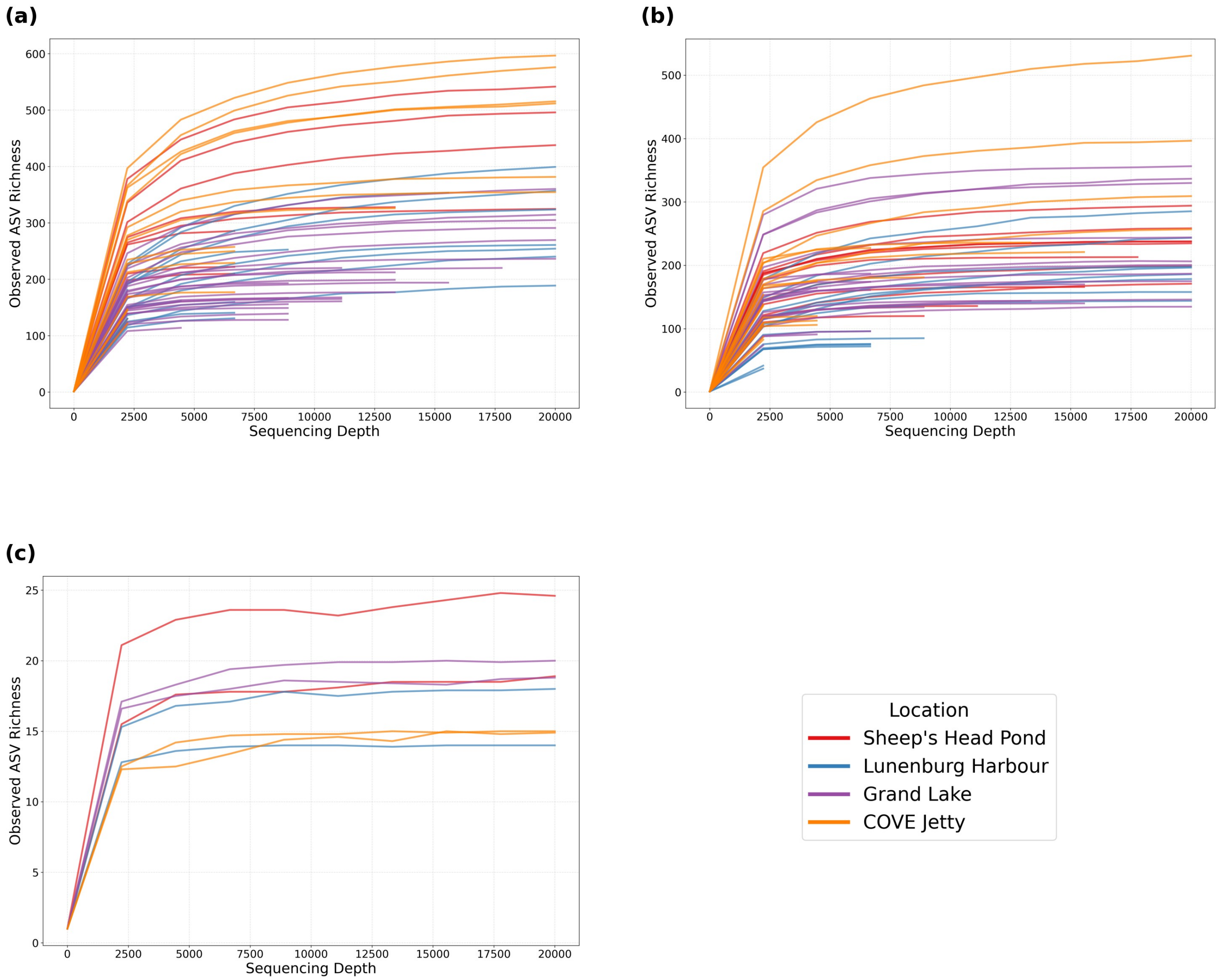

FIGURE S1 Repeated rarefaction curves for (a) 16S, (b) 18S, and (c) 12S sequences. Each point represents the average of ten replicate rarefactions to the corresponding sequence depth on the x-axis.

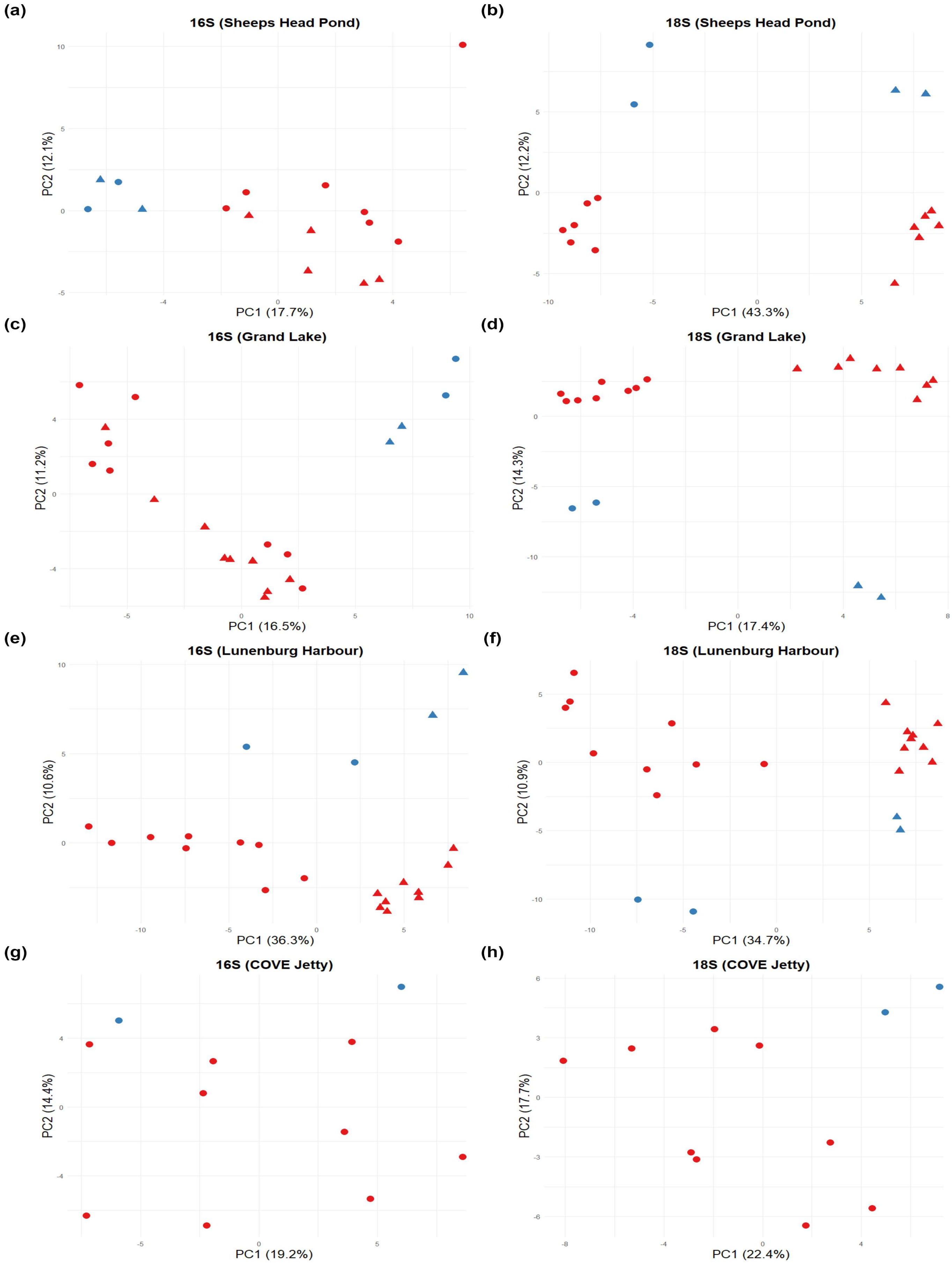

FIGURE S2 Principal coordinate plots based on the Aitchison distance for 16S (a-d) and 18S (e-h) markers for all locations. Circles indicate DOT samples and triangles Niskin samples. Red = DNA, blue = RNA.

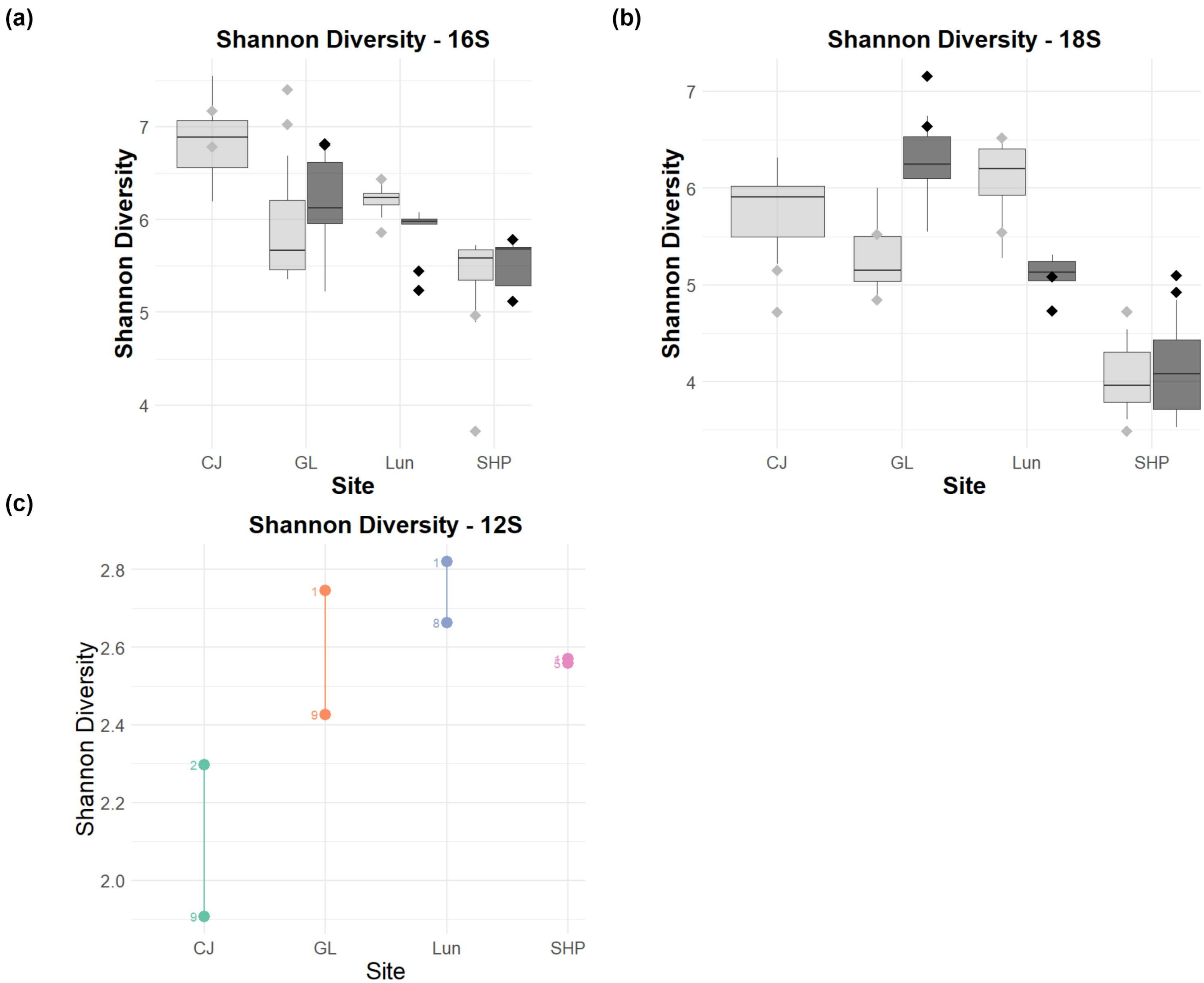

FIGURE S3 Shannon diversity of samples by marker and location. Diversity scores of DNA samples for 16S (a) and 18S (b) are summarized using boxplots, with the two RNA samples shown with diamonds. Light gray boxes = DOT sampler, dark gray = Niskin sampler. No Niskin samples were collected for CJ. (c) Shannon diversity scores of DOT DNA samples for the 12S marker (two per location, DOT and DNA only). Time points are shown and points are coloured by location.

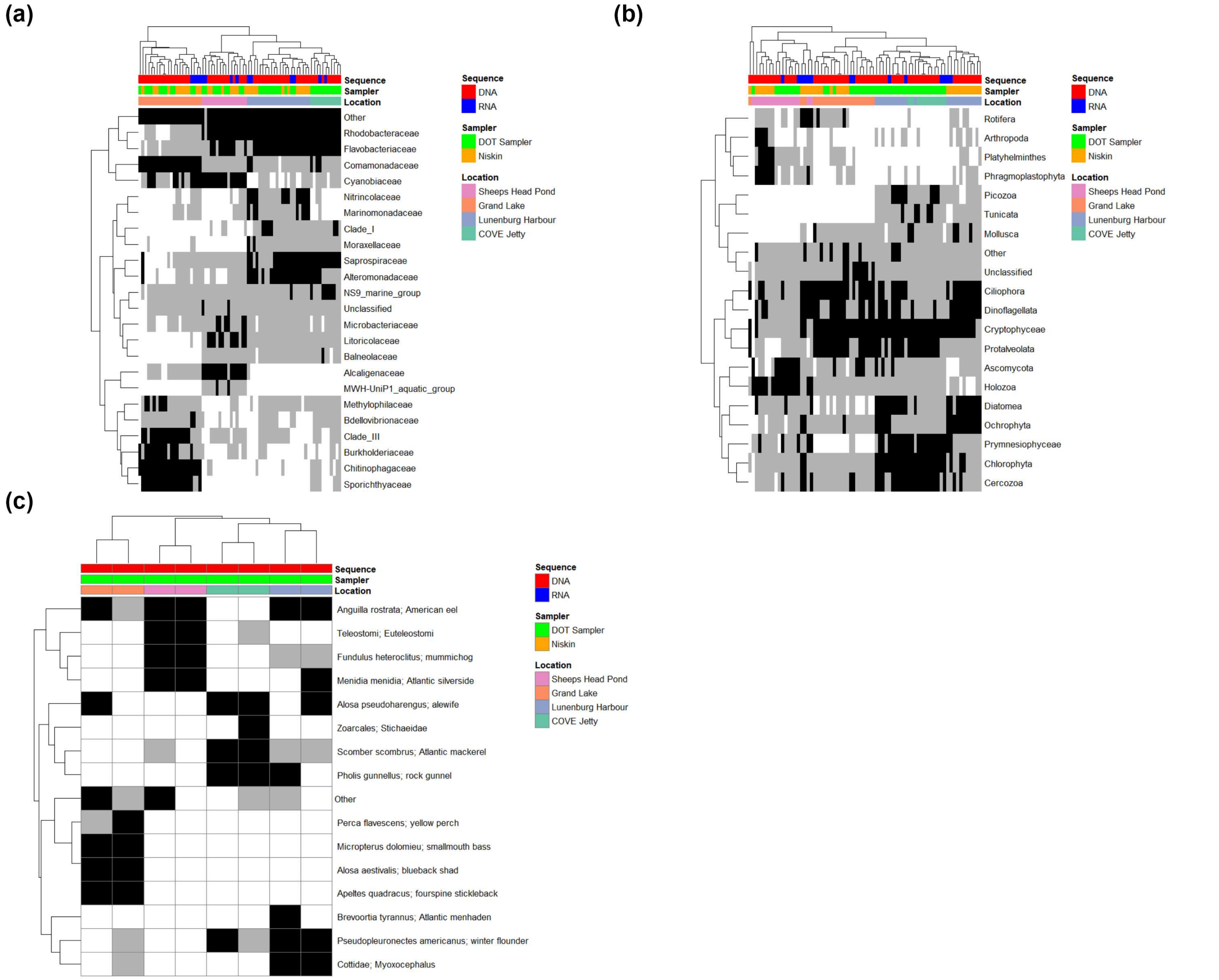

FIGURE S4 Presence / absence heatmaps for all samples by location, sequence type (DNA vs RNA) and sampler (DOT vs Niskin) for 16S families (a), 18S phyla (b), and 12S species (c) profiles. All taxa shown are present in at least one sample at a minimum relative abundance of 5%. Samples with taxon abundance above this threshold are shown are indicated with black rectangles, while taxon abundance $\geq$ 0.1% and < 5% are shown in gray.

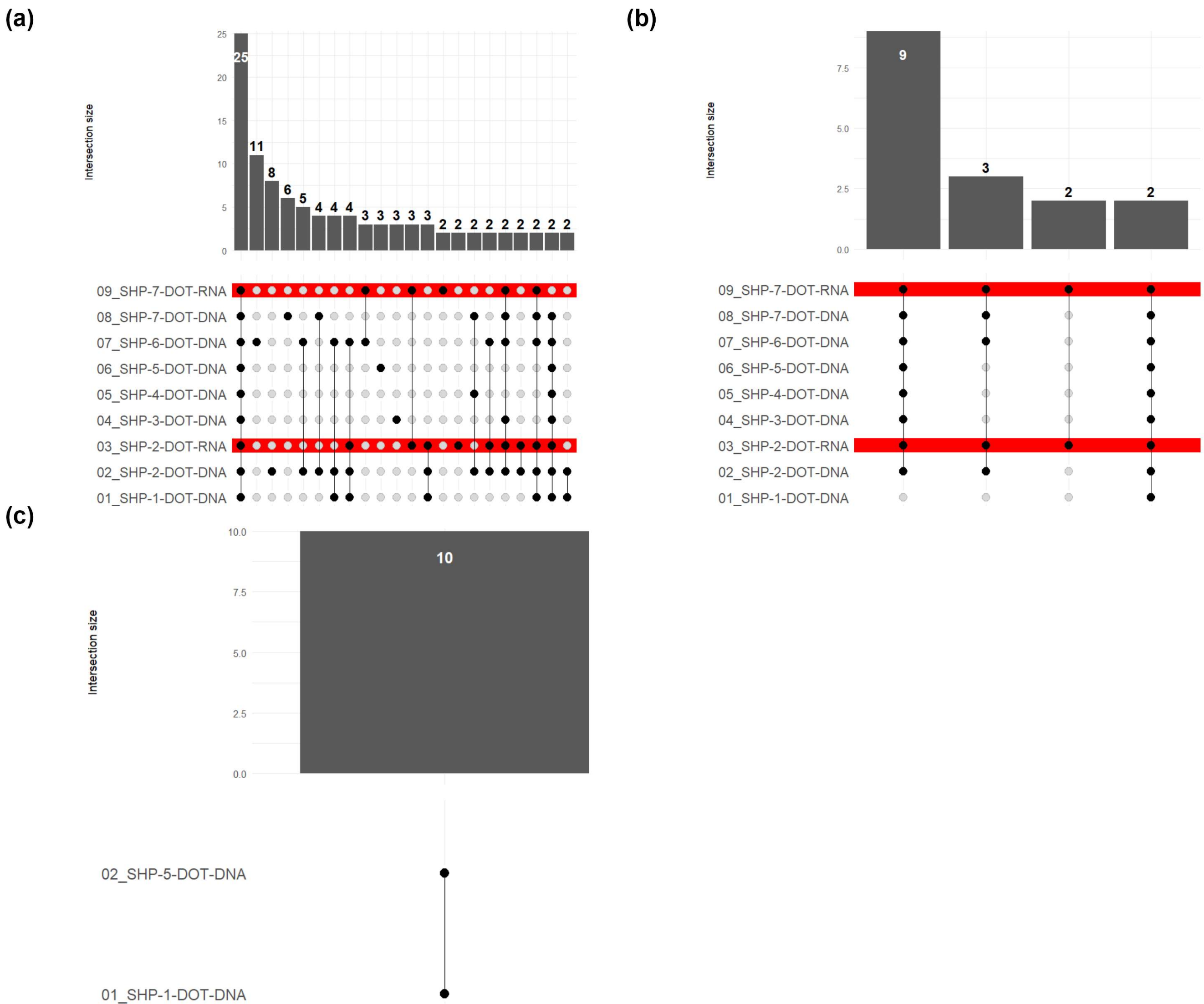

FIGURE S5 UpSet plots showing distribution of taxa across all DOT DNA and RNA samples for 16S families (a), 18S phyla (b), and 12S species (c) profiles in Sheeps Head Pond. Each column in the plot indicates a unique presence / absence pattern across all samples, with black dots indicating presence and white dots absence. Bars at the top with numbers indicate the number of taxa that exhibit that presence/absence pattern. RNA profiles for 16S and 18S are highlighted in red. Numbers preceding each sample name are for ordering purposes only.

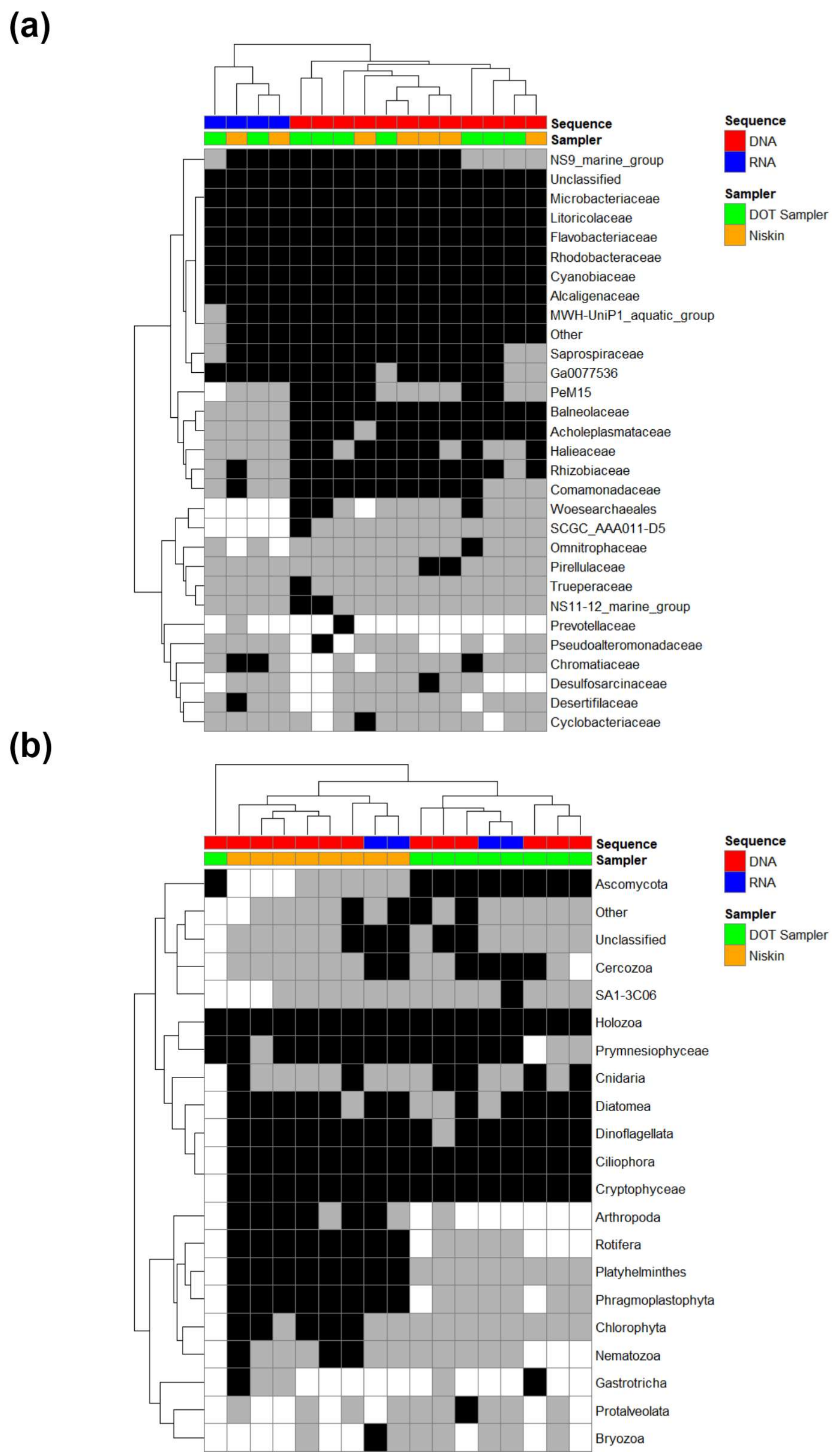

FIGURE S6 Presence / absence heatmaps for all samples by sequence type and sampler for 16S families (a) and 18S phyla (b) from Sheeps Head Pond. All taxa shown are present in at least one sample at a minimum relative abundance of 1%. Samples with taxon abundance above this threshold are shown are indicated with black rectangles, while samples with taxon abundance less than 1% are shown in gray.

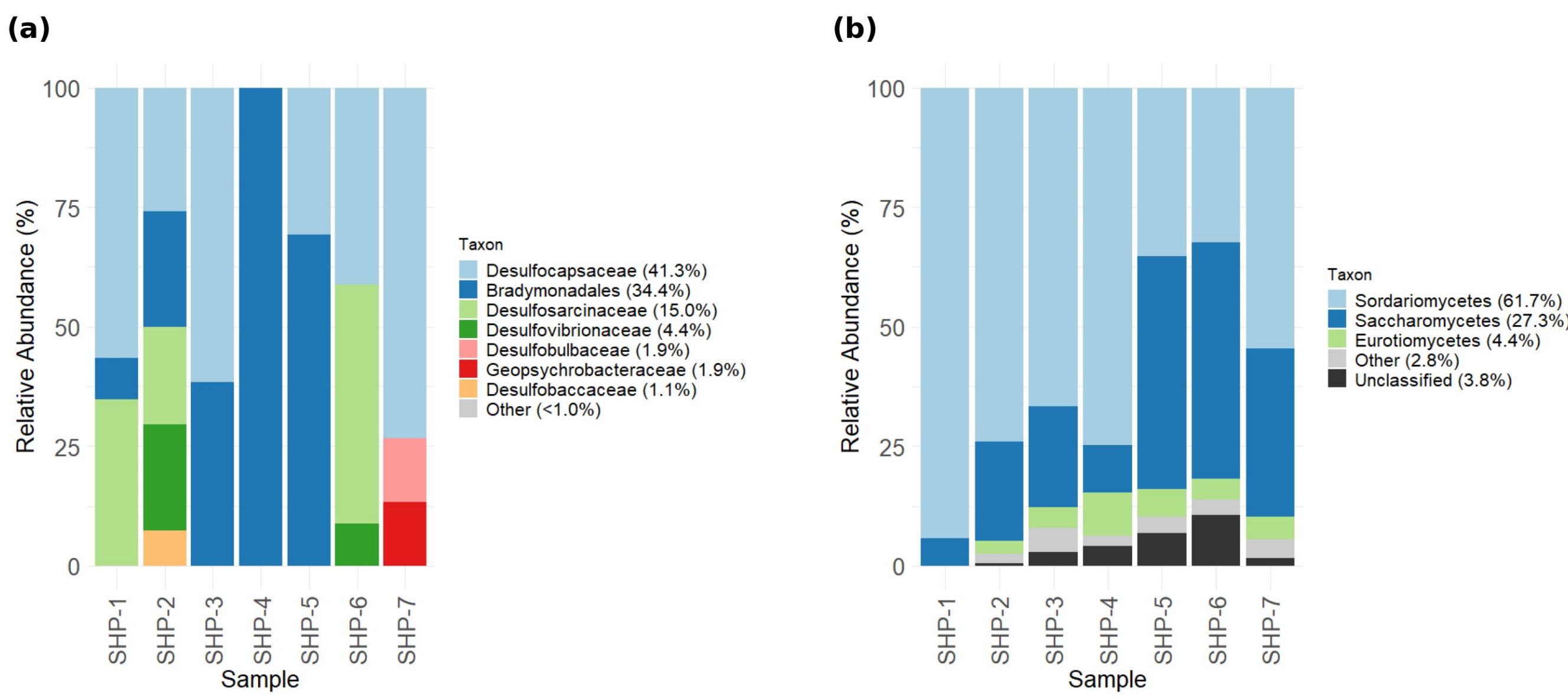

FIGURE S7 Taxonomic distributions of taxonomic classes within the bacterial phylum Desulfobacterota (a) and the eukaryotic phylum Ascomycota (b). Taxa with abundance $\geq$ 1% in at least one sample are shown.

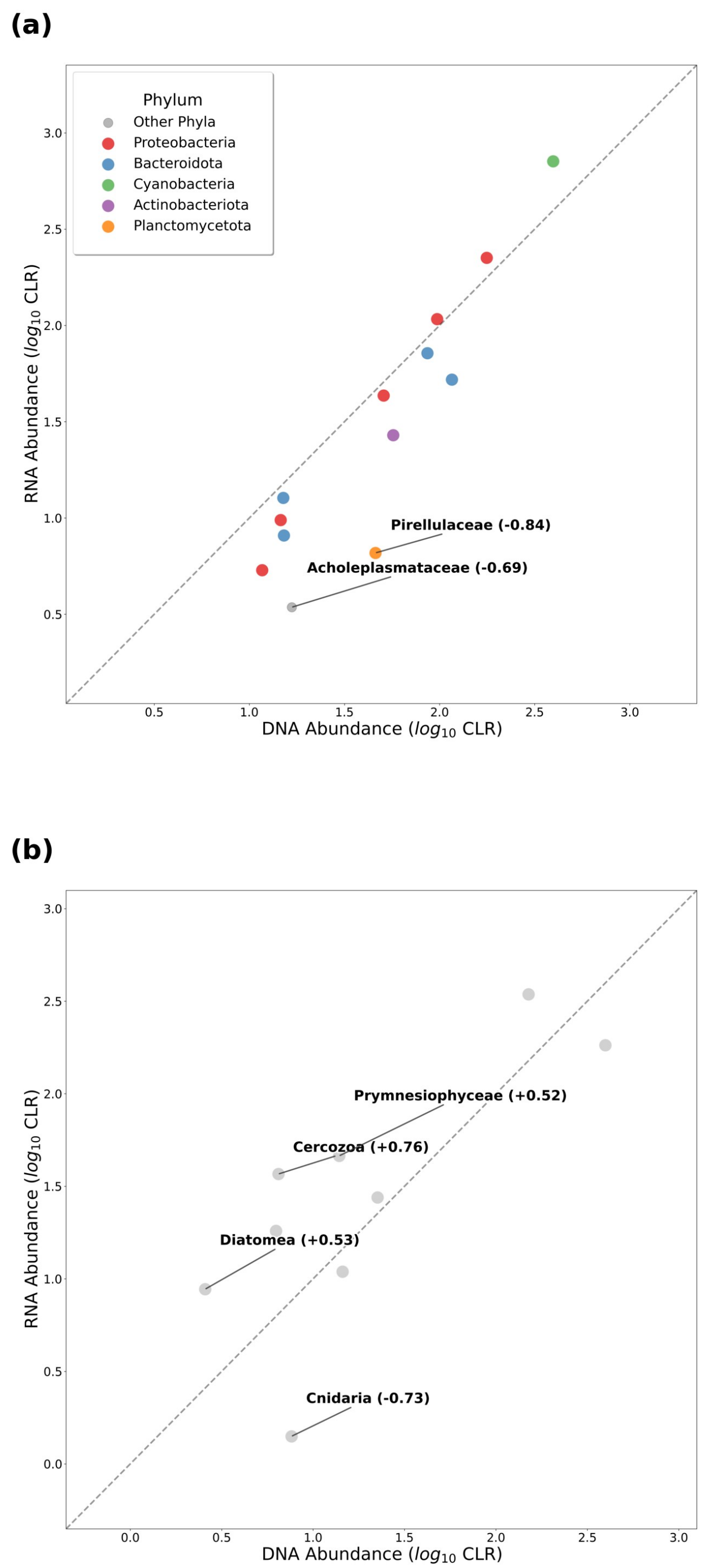

FIGURE S8 Abundance and centered log-ratio comparisons for paired DNA and RNA samples for 16S families (a) and 18S phyla (b) from Sheeps Head Pond. x- and y-axes show the relative abundance of each taxon in the DNA and RNA samples respectively on a logarithmic scale. Families from the five most-abundant 16S phyla are indicated with colours, and families with absolute CLR differences ≥ 0.5 are shown.

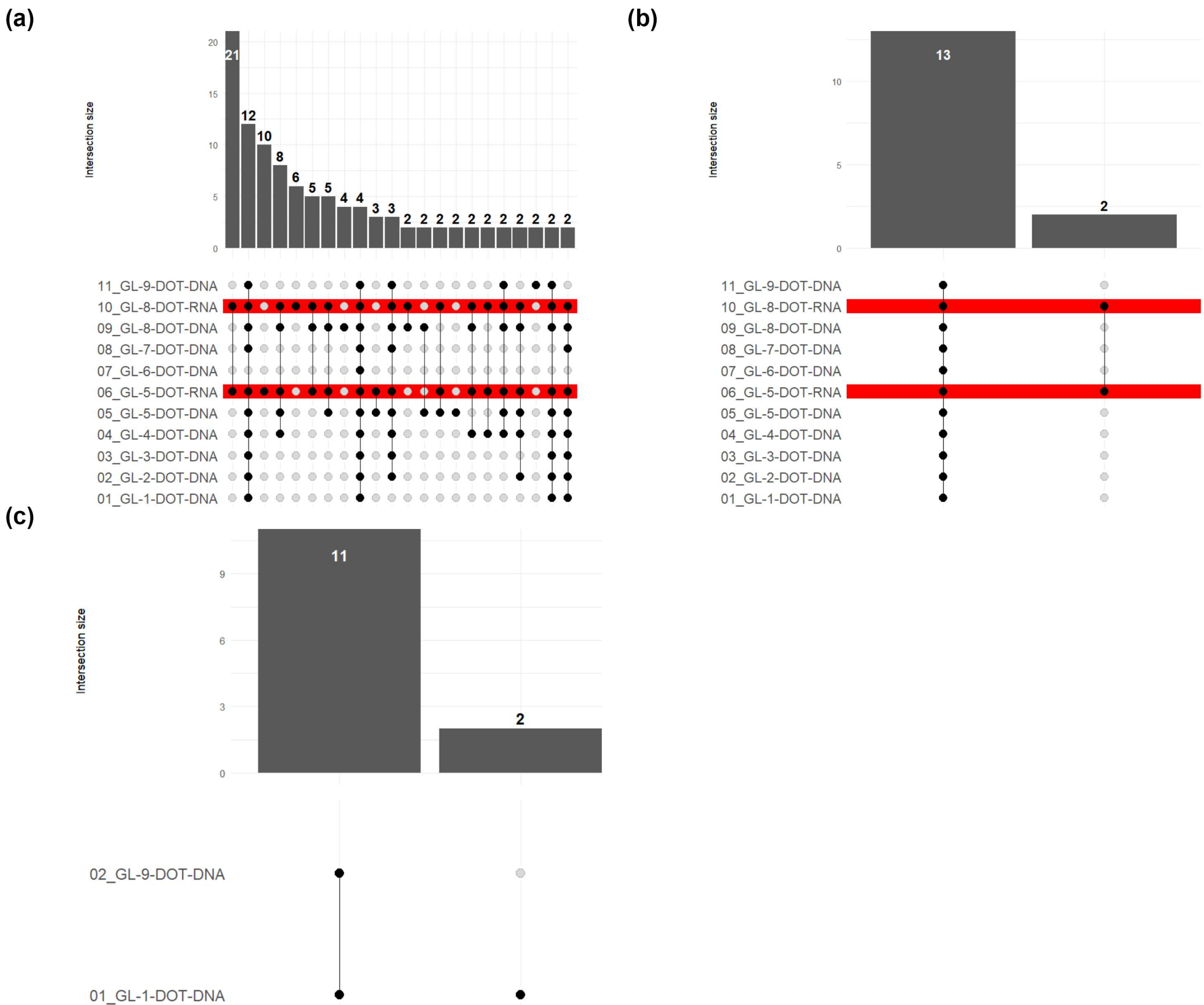

FIGURE S9 UpSet plots showing distribution of taxa across all DOT DNA and RNA samples for 16S families (a), 18S phyla (b), and 12S species (c) profiles in Grand Lake. Each column in the plot indicates a unique presence / absence pattern across all samples, with black dots indicating presence and white dots absence. Bars at the top with numbers indicate the number of taxa that exhibit that presence/absence pattern. RNA profiles for 16S and 18S are highlighted in red. Numbers preceding each sample name are for ordering purposes only.

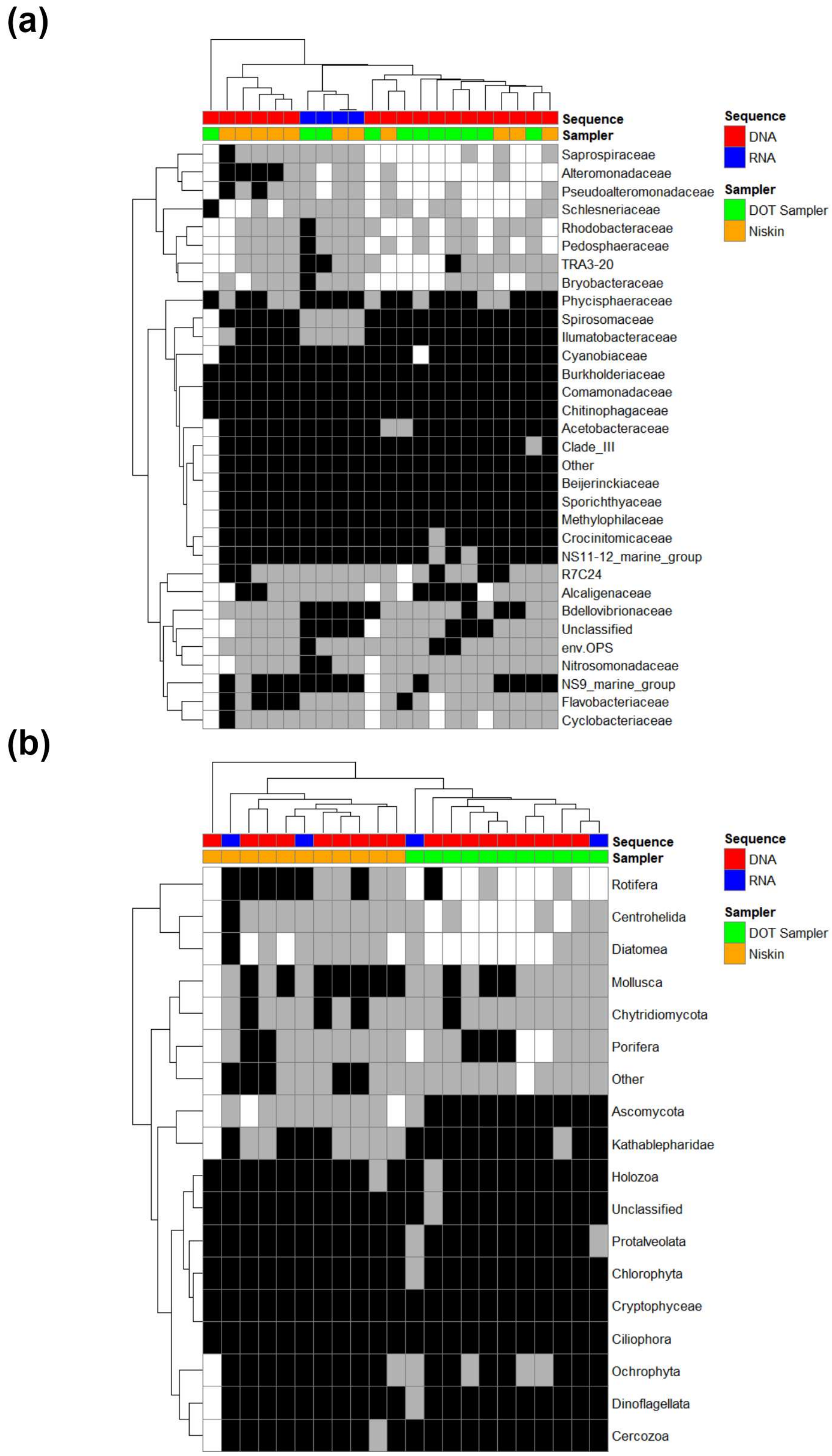

FIGURE S10 Presence / absence heatmaps for all samples by sequence type and sampler for 16S families (a) and 18S phyla (b) from Grand Lake. All taxa shown are present in at least one sample at a minimum relative abundance of 1%. Samples with taxon abundance above this threshold are shown are indicated with black rectangles, while samples with taxon abundance less than 1% are shown in gray.

(a)

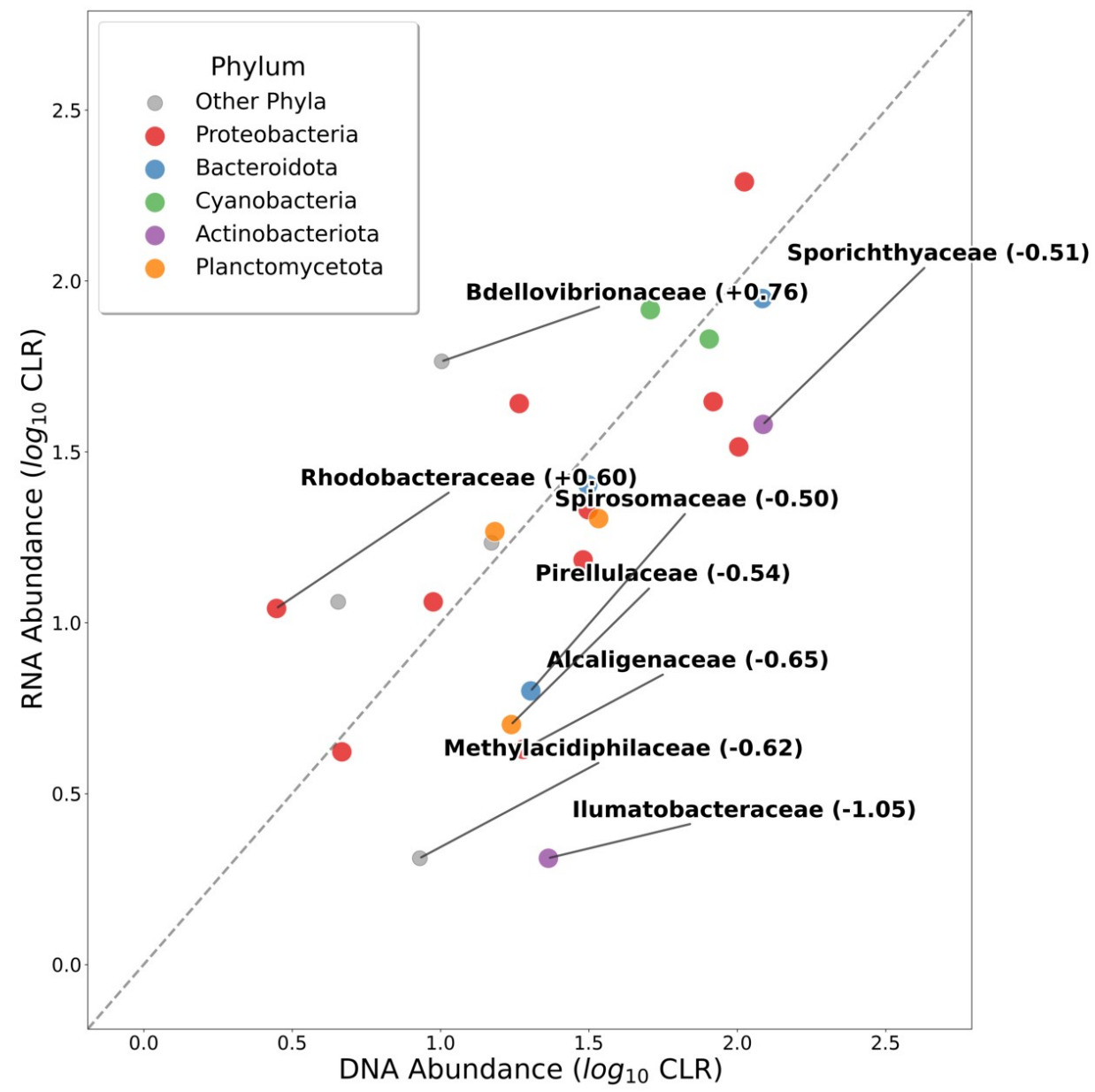

(b)

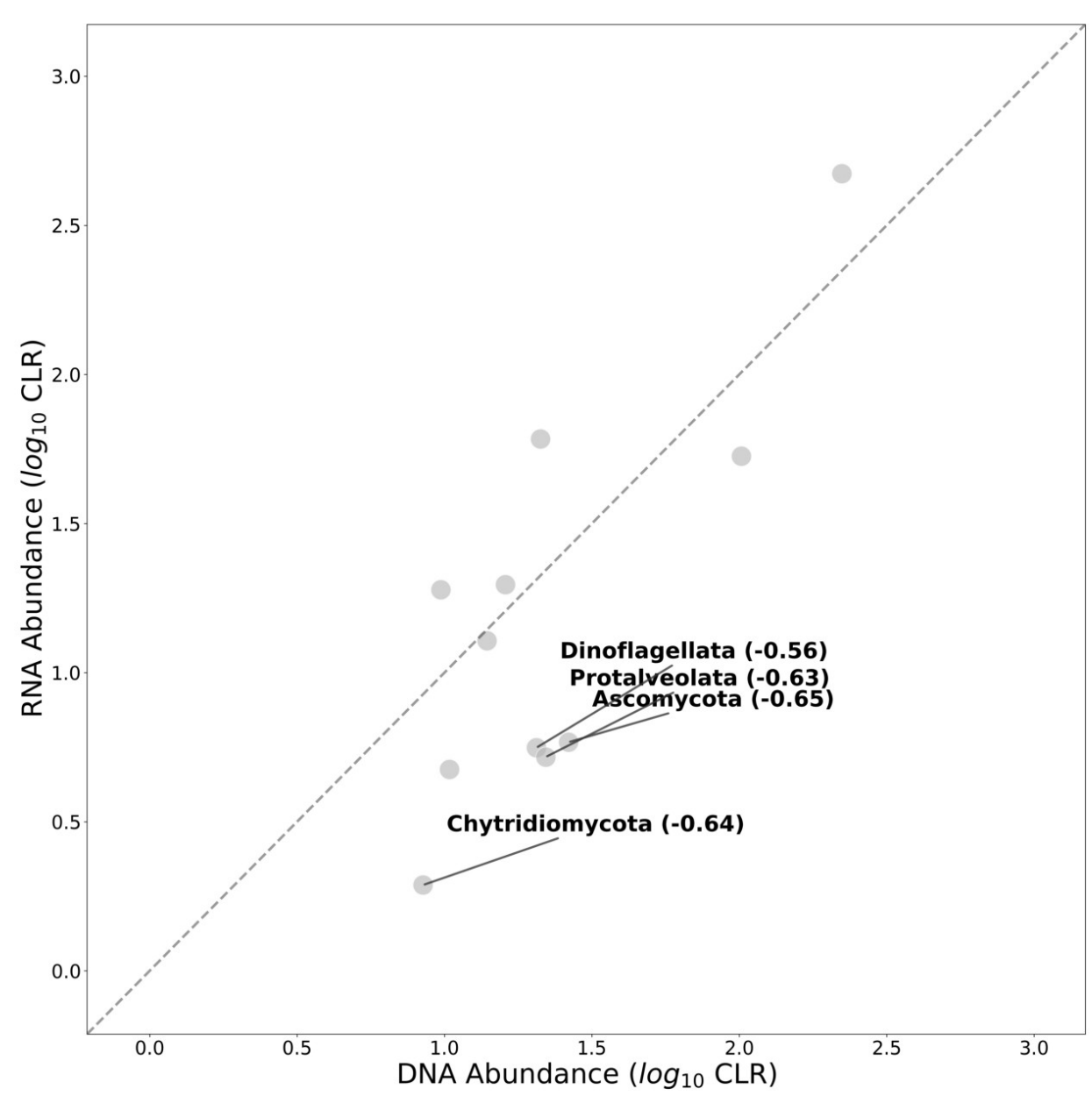

FIGURE S11 Abundance and centered log-ratio comparisons for paired DNA and RNA samples for 16S families (a) and 18S phyla (b) from Grand Lake. x- and y-axes show the relative abundance of each taxon in the DNA and RNA samples respectively on a logarithmic scale. Families from the five most-abundant 16S phyla are indicated with colours, and families with absolute CLR differences $\geq$ 0.5 are shown.

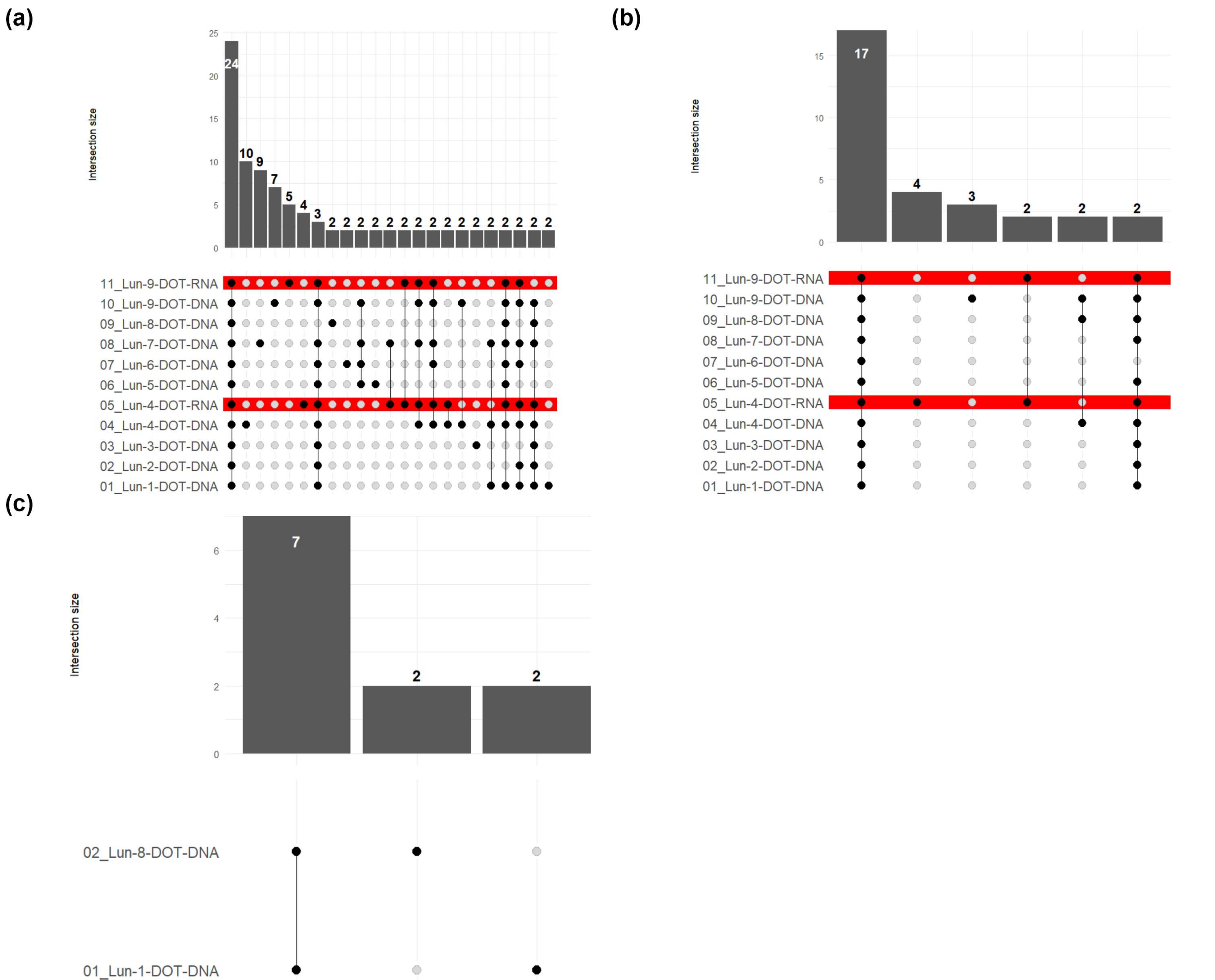

FIGURE S12 UpSet plots showing distribution of taxa across all DOT DNA and RNA samples for 16S families (a), 18S phyla (b), and 12S species (c) profiles in Lunenburg Harbour. Each column in the plot indicates a unique presence / absence pattern across all samples, with black dots indicating presence and white dots absence. Bars at the top with numbers indicate the number of taxa that exhibit that presence/absence pattern. RNA profiles for 16S and 18S are highlighted in red. Numbers preceding each sample name are for ordering purposes only.

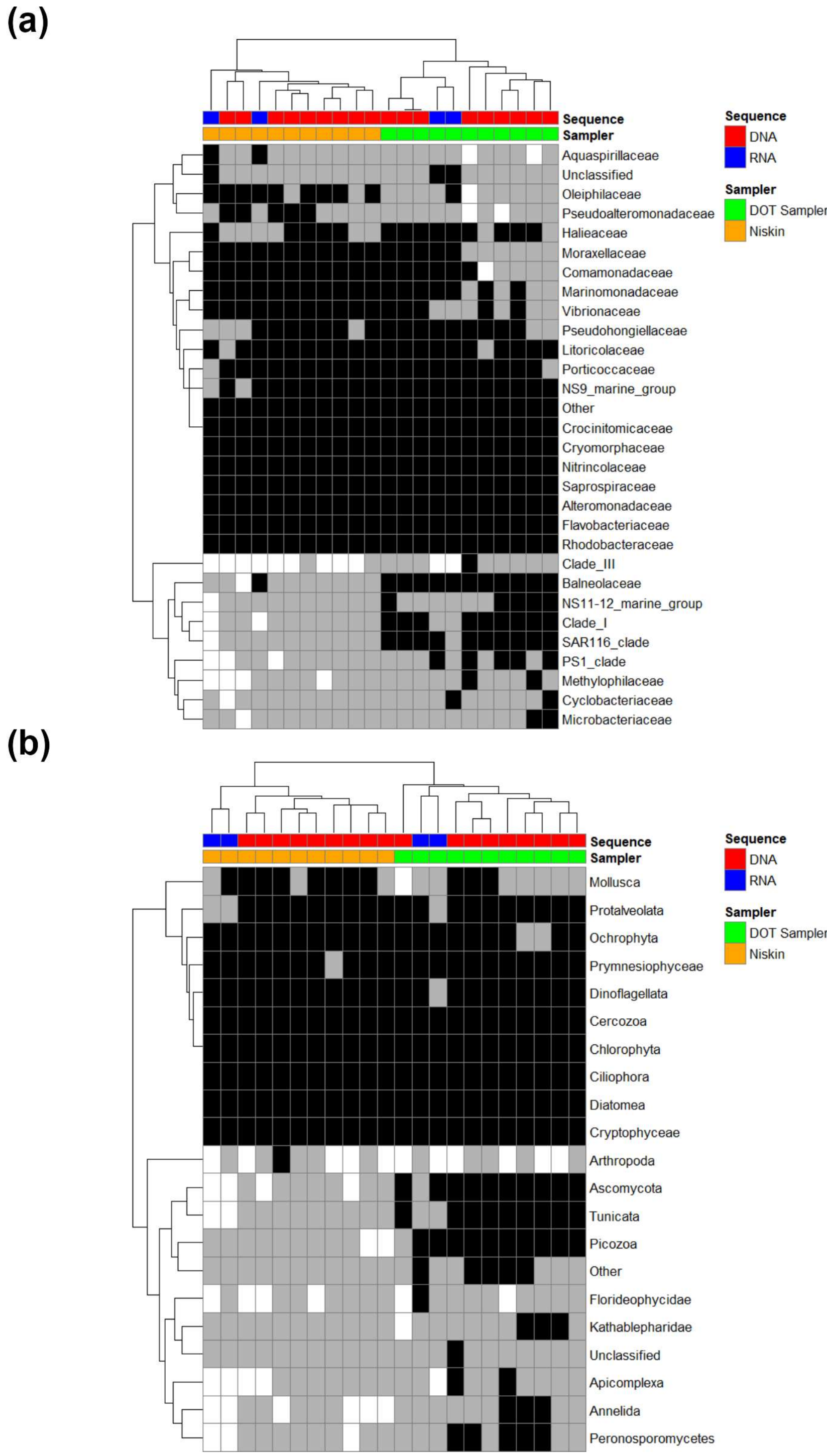

FIGURE S13 Presence / absence heatmaps for all samples by sequence type and sampler for 16S families (a) and 18S phyla (b) from Lunenburg Harbour. All taxa shown are present in at least one sample at a minimum relative abundance of 1%. Samples with taxon abundance above this threshold are shown are indicated with black rectangles, while samples with taxon abundance less than 1% are shown in gray.

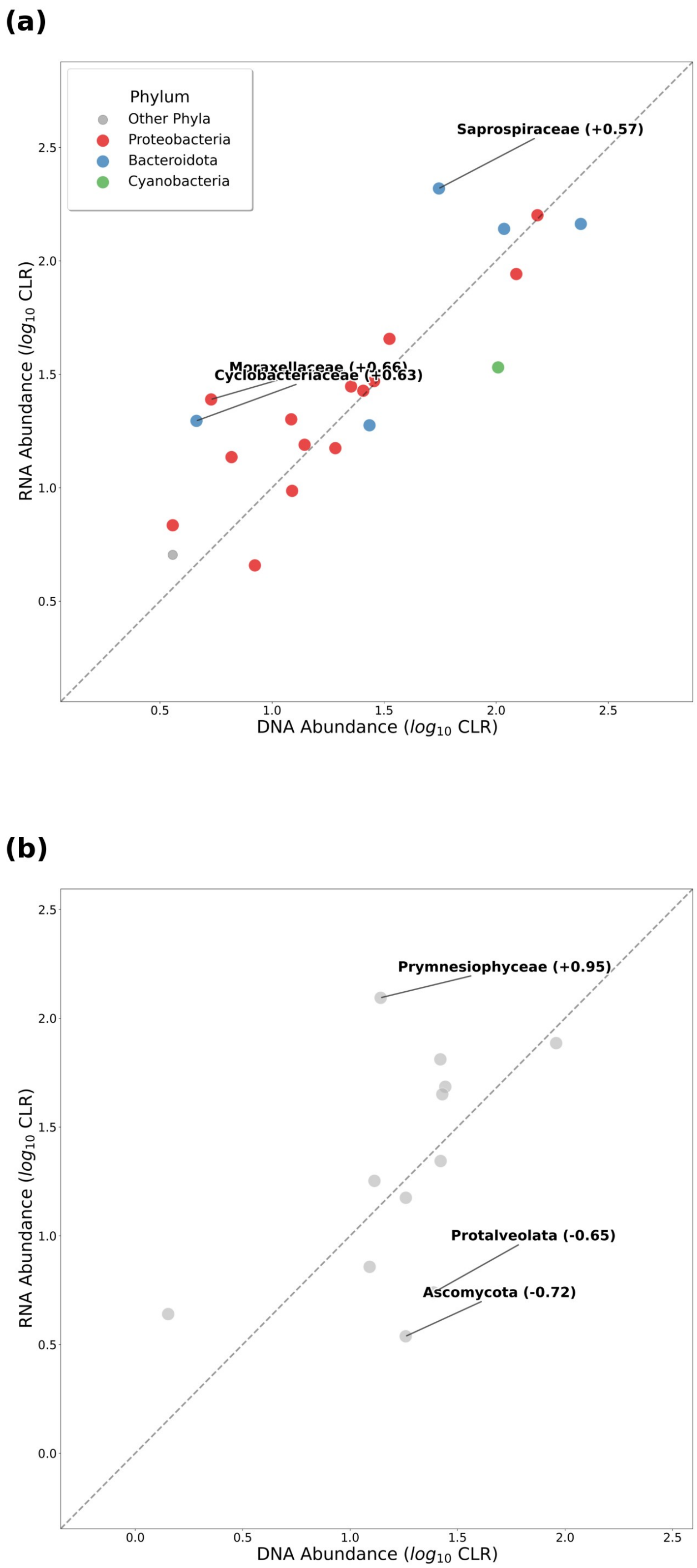

FIGURE S14 Abundance and centered log-ratio comparisons for paired DNA and RNA samples for 16S families (a) and 18S phyla (b) from Lunenburg Harbour. x- and y-axes show the relative abundance of each taxon in the DNA and RNA samples respectively on a logarithmic scale. Families from the five most-abundant 16S phyla are indicated with colours, and families with absolute CLR differences ≥ 0.5 are shown.

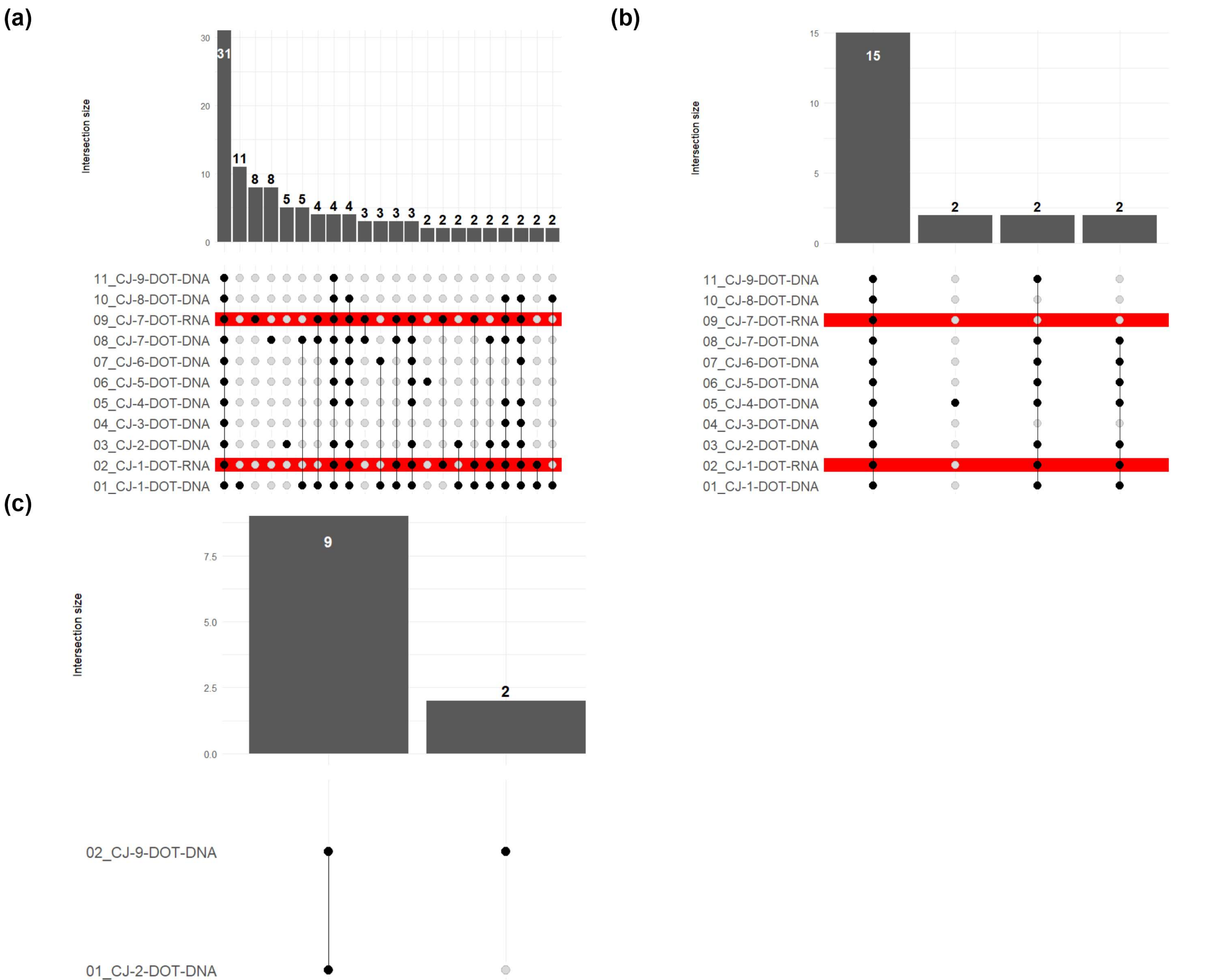

FIGURE S15 UpSet plots showing distribution of taxa across all DOT DNA and RNA samples for 16S families (a), 18S phyla (b), and 12S species (c) profiles from the COVE Jetty in Halifax Harbour. Each column in the plot indicates a unique presence / absence pattern across all samples, with black dots indicating presence and white dots absence. Bars at the top with numbers indicate the number of taxa that exhibit that presence/absence pattern. RNA profiles for 16S and 18S are highlighted in red. Numbers preceding each sample name are for ordering purposes only.

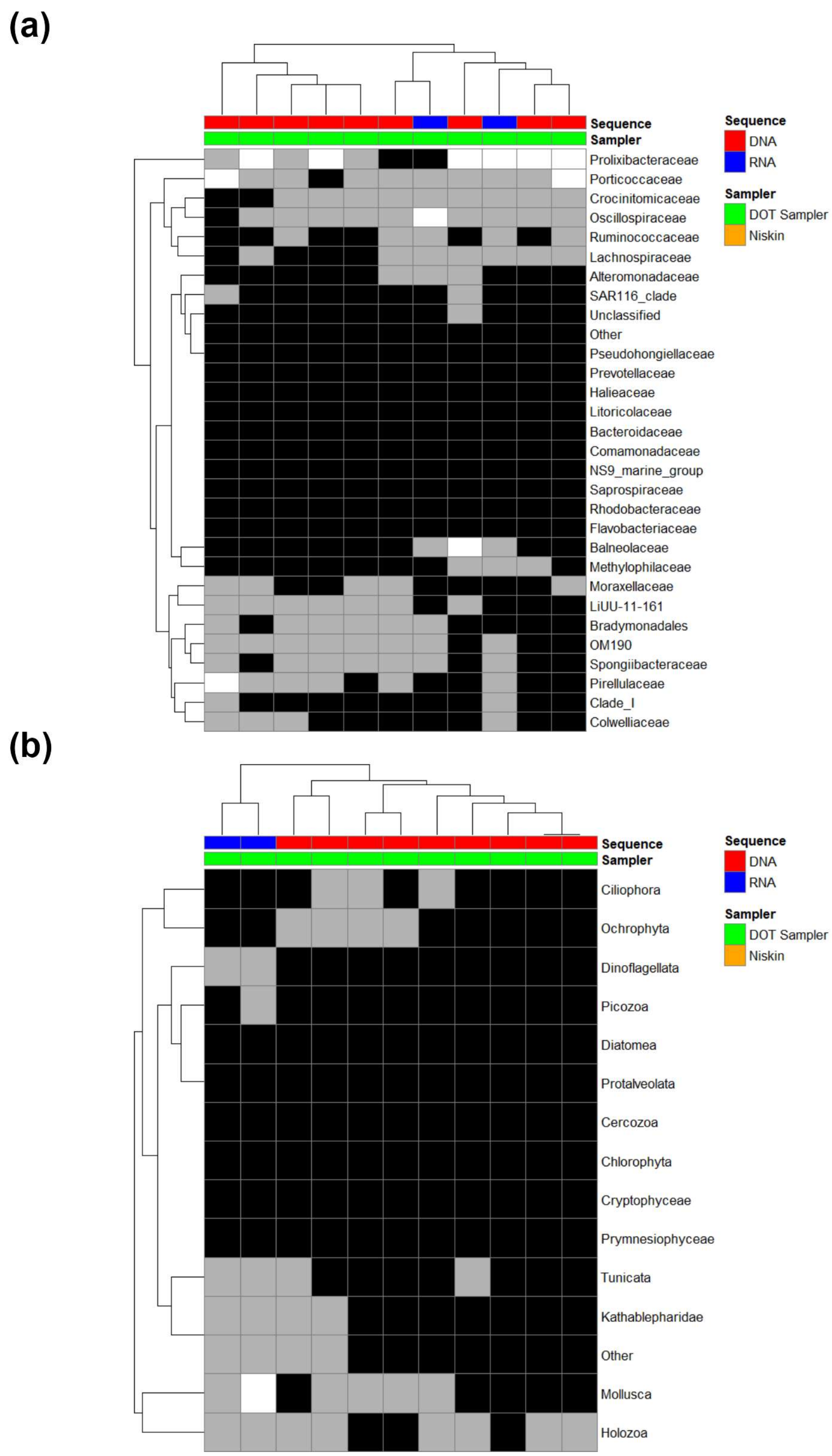

FIGURE S16 Presence / absence heatmaps for all samples by sequence type and sampler for 16S families (a) and 18S phyla (b) from COVE Jetty. All taxa shown are present in at least one sample at a minimum relative abundance of 1%. Samples with taxon abundance above this threshold are shown are indicated with black rectangles, while samples with taxon abundance less than 1% are shown in gray.

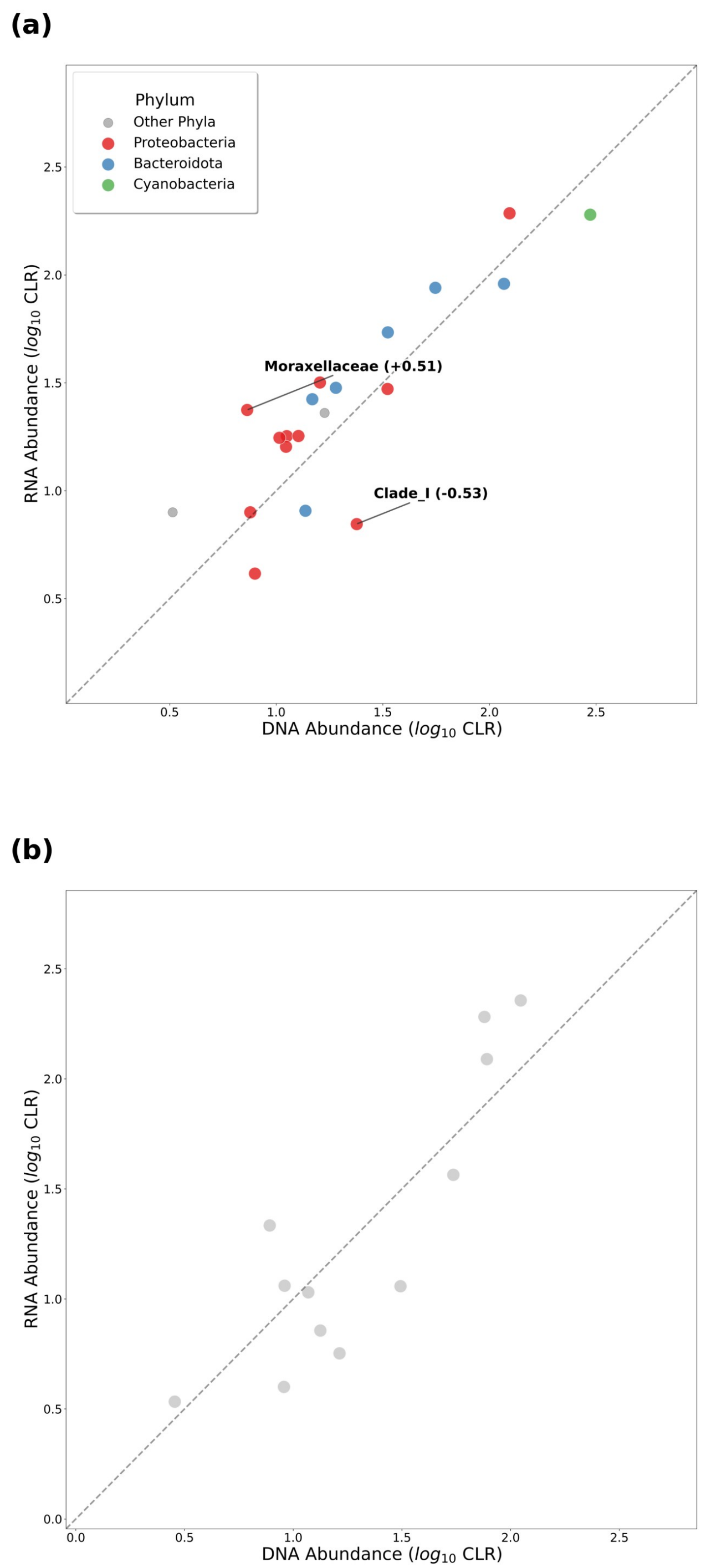

FIGURE S17 Abundance and centered log-ratio comparisons for paired DNA and RNA samples for 16S families (a) and 18S phyla (b) from COVE Jetty. x- and y-axes show the relative abundance of each taxon in the DNA and RNA samples respectively on a logarithmic scale. Families from the five most-abundant 16S phyla are indicated with colours, and families with absolute CLR differences ≥ 0.5 are shown.

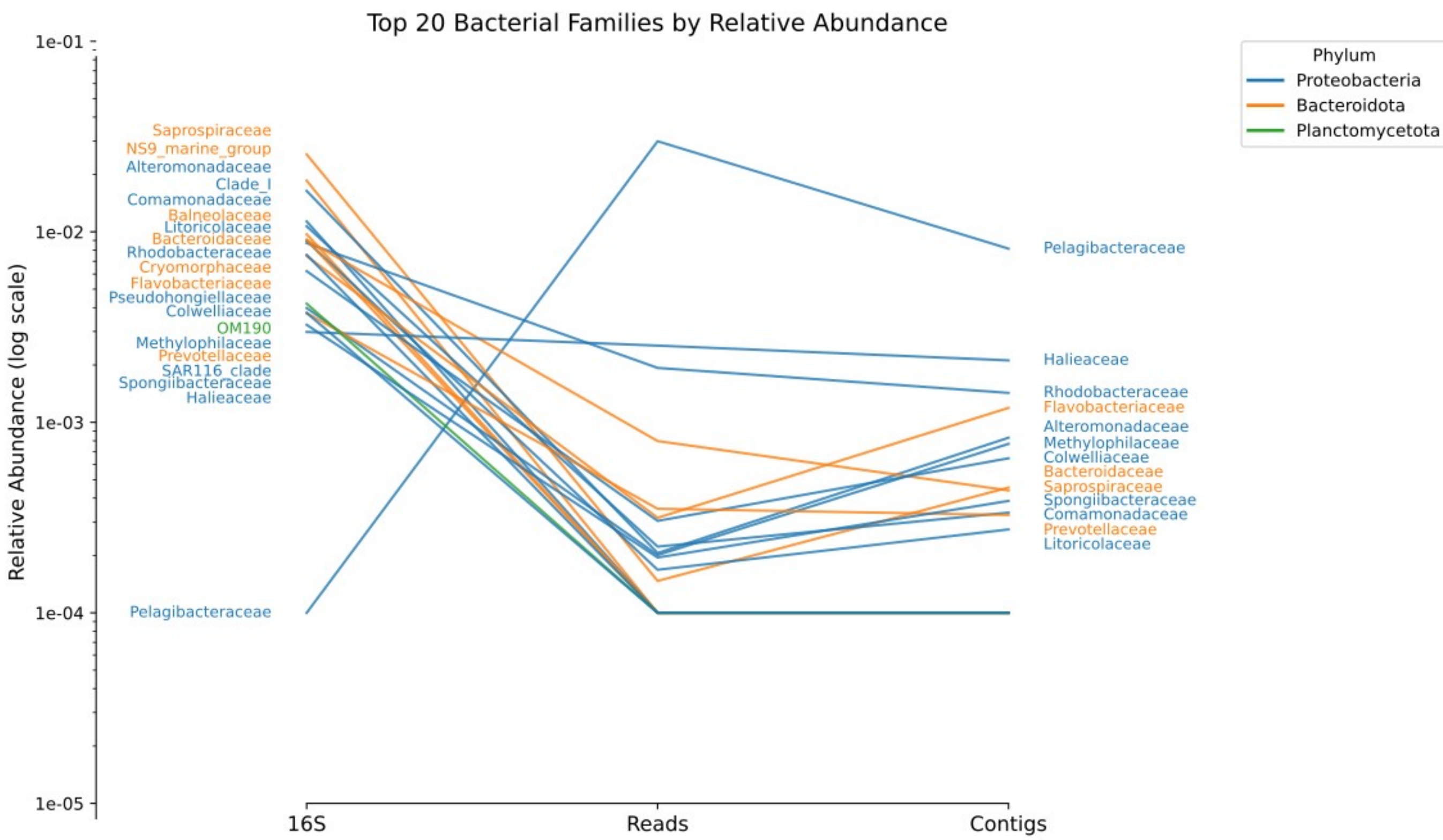

FIGURE S18 Abundance profiles for the 20 most-abundant prokaryotic families inferred from 16S marker-gene and metagenomic reads and contigs from the COVE Jetty in Halifax Harbour. The y-axis indicates the relative abundance of a given family in each of the different sample types on a logarithmic scale. Colours indicate the phylum of each identified family.

TABLE S1 Summary of samples, sequencing metrics, and metadata (Table S1).

Location and Date	Sample Name	Method	Sample start time	Preservation time	Volume (mL)	[DNA] ng/ÂµL	16S DNA Reads	16S cDNA Reads	18S DNA Reads	18S cDNA Reads	12S DNA Reads	MG Reads
	SHP-1-DOT	DOT	9:32	9:43	27	27.4	8698		179			63387
	SHP-2-DOT	DOT	10:32	10:44	51	23.1	34313	93332	39910	118160		
	SHP-3-DOT	DOT	11:32	11:44	33	18.0	13453		12495			
	SHP-4-DOT	DOT	12:32	12:41	27	21.2	21598		4873			
Sheep’s Head Pond	SHP-5-DOT	DOT	13:32	13:45	45	19.9	19973		3466			61237
SHP	SHP-6-DOT	DOT	14:32	14:45	39	15.8	17275		9118			
Tantallon, NS	SHP-7-DOT	DOT	15:32	15:44	54	8.1	36305	103447	27390	119807		
44Â°39’58.0”N	SHP-1-NIS	Niskin	9:30	10:00	42	29.4	14255		2138			
63Â°54’02.3”W	SHP-2-NIS	Niskin	10:30	10:45	50	17.9	26773	84185	28095	113134		
July 17, 2023	SHP-3-NIS	Niskin	11:43	11:55	43	15.8	11718		11381			
	SHP-4-NIS	Niskin	12:35	12:47	39	18.8	1		0			
	SHP-5-NIS	Niskin	14:40	14:48	35	13.5	1		9689			
	SHP-6-NIS	Niskin	13:43	13:50	42	13.2	6132		8964			
	SHP-7-NIS	Niskin	15:34	15:43	43	5.3	32352	101011	32339	119403		
	GL-1-DOT	DOT	9:47	9:57	30	10.1	4166		5439			79331
	GL-2-DOT	DOT	10:32	10:38	39	12.1	5358		2620			
	GL-3-DOT	DOT	11:17	11:23	42	5.8	2827		6435			
	GL-4-DOT	DOT	12:02	12:08	39	12.1	6273		5658			
	GL-5-DOT	DOT	12:47	12:54	45	4.0	10510	84112	7235	119537		
	GL-6-DOT	DOT	13:32	13:39	48	9.4	188		5482			
Grand Lake	GL-7-DOT	DOT	14:17	14:23	39	12.8	4532		2911			
GL	GL-8-DOT	DOT	15:02	15:08	42	5.4	13027	74091	12483	107014		
Oakfield, NS	GL-9-DOT	DOT	15:47	15:53	42	7.7	4603		1495		15814	82018
44Â°54’18.9”N	GL-1-NIS	Niskin	9:50	10:03	52	4.8	4578		322			
63Â°35’55.3”W	GL-2-NIS	Niskin	10:33	10:40	63	8.4	56188		26372			
July 21, 2023	GL-3-NIS	Niskin	11:23	11:30	50	6.5	16369		8778			
	GL-4-NIS	Niskin	12:07	~12:11	52	6.8	58019		25225			
	GL-5-NIS	Niskin	13:13	13:17	43	3.4	11163	92666	5780	79018		
	GL-6-NIS	Niskin	13:46	13:51	50	2.0	4270		5456			
	GL-7-NIS	Niskin	14:30	14:38	55	2.9	6985		5479			
	GL-8-NIS	Niskin	15:21	15:29	53	5.6	20085	95373	11833	95019		
	GL-9-NIS	Niskin	15:57	16:02	50	5.2	4985		10005			
	Lun-1-DOT	DOT	9:32	9:45	125	23.7	25389		11293			70281
	Lun-2-DOT	DOT	10:17	10:28	125	13.6	21944		12334			
	Lun-3-DOT	DOT	11:02	11:17	123	31.3	22824		9696			
	Lun-4-DOT	DOT	11:47	12:02	120	14.6	41685	79337	33415	78848		
	Lun-5-DOT	DOT	12:32	12:44	84	39.2	26884		11790			
	Lun-6-DOT	DOT	13:17	13:27	69	42.1	23519		5789			
ABCO Dock	Lun-7-DOT	DOT	14:02	14:15	96	29.0	33706		4910			
Lunenburg, NS	Lun-8-DOT	DOT	14:47	14:57	72	31.4	17832	4927	30589	79258		
Lun	Lun-9-DOT	DOT	15:32	15:43	78	13.2	37522	71379	35630	46355		
44Â°22’31.4”N	Lun-1-NIS	Niskin	9:39	9:58	30	111.9	34837		8865			
64Â°19’01.3”W	Lun-2-NIS	Niskin	10:18	10:26	40	39.7	25919		12398			
July 19, 2023	Lun-3-NIS	Niskin	11:12	11:18	20	36.2	32142		37540			
	Lun-4-NIS	Niskin	11:57	12:06	19	8.0	35429	13581	37634	44050		
	Lun-5-NIS	Niskin	12:35	12:40	28	38.0	28578		22580			
	Lun-6-NIS	Niskin	13:33	13:39	26	30.0	16139		23225			
	Lun-7-NIS	Niskin	14:20	14:25	30	29.0	35428		17222			
	Lun-8-NIS	Niskin	14:57	15:03	28	24.4	21418		21344			
	Lun-9-NIS	Niskin	15:41	15:49	31	4.4	21274	18502	34816	43868		
	CJ-1-DOT	DOT	17:27	17:41	125	21.1	44065	75370	58755	121905		14694509
COVE Jetty	CJ-2-DOT	DOT	20:02	20:15	96	39.6	14113		41949		80441	14968251
CJ	CJ-3-DOT	DOT	0:02	0:12	57	20.4	7688		15147			15362084
Dartmouth, NS	CJ-4-DOT	DOT	4:02	4:19	125	24.1	23424		43986			15863813
44Â°39’38.7”N	CJ-5-DOT	DOT	8:02	8:12	125	15.6	6635		35532			21703405
63Â°33’29.4”W	CJ-6-DOT	DOT	12:02	12:11	125	12.1	18530		32185			16692714
July 22–24, 2023	CJ-7-DOT	DOT	16:02	16:13	84	16.2	50705	87261	62740	138934		16655421
	CJ-8-DOT	DOT	20:02	20:16	111	45.4	18208		20513			15297148
	CJ-9-DOT	DOT	0:02	0:15	125	44.0	9227		29544		66223	18890813

TABLE S2 Statistics for MegaHIT assemblies (M1â€"M9). Genus names are based on Kraken2 annotations.

**MegaHIT Assemblies**	**M1**	**M2**	**M3**	**M4**	**M5**	**M6**	**M7**	**M8**	**M9**
# contigs ($\geq$ 0 bp)	6054	6657	6215	5549	6200	4336	4983	6149	7939
# contigs ($\geq$ 1000 bp)	6054	6657	6215	5549	6200	4336	4983	6149	7939
# contigs ($\geq$ 5000 bp)	1611	1567	1681	1261	1275	876	1210	1308	1385
# contigs ($\geq$ 10000 bp)	492	562	615	418	422	329	478	524	460
# contigs ($\geq$ 25000 bp)	104	134	156	108	156	120	143	164	121
# contigs ($\geq$ 50000 bp)	43	43	60	36	45	37	55	50	44
Total length ($\geq$ 0 bp)	31839659	35370067	37064402	28357715	31661455	22775388	29411453	32988181	36603097
Total length ($\geq$ 1000 bp)	31839659	35370067	37064402	28357715	31661455	22775388	29411453	32988181	36603097
Total length ($\geq$ 5000 bp)	18181451	20208360	23608131	15591115	17055547	12673295	18425537	18962901	17607163
Total length ($\geq$ 10000 bp)	10625488	13419536	16304110	9860477	11395816	9059928	13419442	13663354	11480390
Total length ($\geq$ 25000 bp)	5127757	7036005	9407010	5278395	7422923	5944302	8402881	8342435	6310551
Total length ($\geq$ 50000 bp)	3009817	3940030	6193784	2869632	3660699	3040108	5348864	4339072	3756839
# contigs	6054	6657	6215	5549	6200	4336	4983	6149	7939
Largest contig	129130	194311	415927	156220	248731	177987	381104	200017	210960
Largest contig Kraken2	*Planktomarina*	*Planktomarina*	*Planktomarina*	*Pseudomonas*	*Flavobacterium*	*Flavobacterium*	*Alteromonas*	*Pseudomonas*	*Planktomarina*
Total length	31839659	35370067	37064402	28357715	31661455	22775388	29411453	32988181	36603097
GC (%)	42.73	44.05	44.86	44.49	43.02	42.34	40.11	45.41	45.22
N50	6010	6250	8016	5736	5623	5978	8350	6631	4749
N90	2532	2470	2541	2448	2423	2423	2488	2428	2365
auN	15112.8	20687.5	32695.7	17322.3	20374.1	22154.2	32395.5	23034.3	17736.9
L50	1197	1111	864	998	1044	641	620	878	1528
L90	4632	5048	4527	4254	4756	3287	3636	4630	6237
# N's per 100 kbp	0	0	0	0	0	0	0	0	0

Marker	Location	Variable	PERMANOVA R2	PERMANOVA p	Permdisp
16S	Sheeps Head Pond	DNA vs RNA	0.152	0.001	0.082
16S	Sheeps Head Pond	Sampler	0.082	0.104	0.803
16S	Lunenburg Harbour	Sampler	0.282	0.001	0.196
16S	Lunenburg Harbour	DNA vs RNA	0.111	0.022	0.318
16S	Grand Lake	Sampler	0.075	0.022	0.014
16S	Grand Lake	DNA vs RNA	0.14	0.001	0.025
16S	COVE Jetty	DNA vs RNA	0.113	0.069	0.039
18S	Sheeps Head Pond	Sampler	0.426	0.001	0.5
18S	Sheeps Head Pond	DNA vs RNA	0.116	0.079	0.091
18S	Lunenburg Harbour	Sampler	0.32	0.001	0.001
18S	Lunenburg Harbour	DNA vs RNA	0.09	0.044	0.113
18S	Grand Lake	Sampler	0.166	0.001	0.008
18S	Grand Lake	DNA vs RNA	0.132	0.001	0.121
18S	COVE Jetty	DNA vs RNA	0.181	0.017	0.001

TABLE S3 PERMANOVA statistics for within-site PCoA analyses.

Family	M1	M2	M3	M4	M5	M6	M7	M8	M9	Total
Actinomycetales		2	1					1	1	5
Alteromonadaceae		2	2				2	2	3	11
Enterobacteriaceae			1		2	1	1			5
Flavobacteriaceae	1	2	2		2		1	2	1	11
Gammaproteobacteria			1	1		1	1			4
Haliaceae					1					1
Litorivicinaceae	1	1	1	1	1	1	1	1	1	9
Micrococcales		1	1				1	1		4
NA					1	1				2
Pelagibacteriaceae	3	1	3	1	1	1	3	1	3	17
Rhodobacteraceae			1			1	1			3
Saprospiraceae	2		1	1	3	2				9
Vibrionaceae		1	1				1	1		4
Total	7	10	15	4	11	8	12	9	9	85

TABLE S4 Distribution of families across samples M1–M9.

Family	M1	M2	M3	M4	M5	M6	M7	M8	M9
Actinomycetales		44.6	51.6					51.6	51.6
Alteromonadaceae		61.0	34.5				34.1	34.4	52.0
Enterobacteriaceae			100.0		100.0	100.0	100.0		
Flavobacteriaceae	36.1	33.4	33.2		35.1		31.3	33.0	38.5
Gammaproteobacteria			40.8	40.6		40.6	40.6		
Haliaceae					41.1				
Litorivicinaceae	36.5	36.5	36.5	36.5	36.5	36.5	36.5	36.5	36.5
Micrococcales		32.1	31.8				32.6	31.8	
NA					33.8	33.8			
Pelagibacteriaceae	39.0	37.5	39.3	37.5	37.5	37.5	39.3	37.5	39.6
Rhodobacteraceae			33.9			33.9	33.9		
Saprospiraceae	33.9		31.2	31.2	36.6	39.4			
Vibrionaceae		86.7	86.7				86.7	86.7	

TABLE S5 Relative abundances of families across samples M1–M9.

Class	M1	M2	M3	M4	M5	M6	M7	M8	M9	Total
disinfecting agents and antiseptics	4	2	5	3	2	3	5	2	4	30
elfamycin		1						1	2	4
fluoroquinolone; diaminopyrimidine; phenicol		1	1			1	1			4
fluoroquinolone; tetracycline						1				1
glycopeptide	3	5	7	1	5	3	5	5	3	37
macrolide					1	1				2
macrolide; streptogramin			1		1		1			3
rifamycin		1	1				1	1		4
tetracycline					1	1				2
Total	7	10	15	4	11	8	12	9	9	85

TABLE S6 Distribution of resistance classes across samples M1–M9.

Family	M1	M2	M3	M4	M5	M6	M7	M8	M9
disinfecting agents and antiseptics	38.34	36.98	39.03	38.18	36.98	38.18	38.98	36.98	38.82
elfamycin		87.02						87.02	
fluoroquinolone; diaminopyrimidine; phenicol		86.67	86.67			86.67	86.67		
fluoroquinolone; tetracycline						41.07			
glycopeptide	34.61	34.29	33.19	31.20	36.03	37.53	33.21	33.32	35.83
macrolide					100.00	100.00			
macrolide; streptogramin			100.00		100.00		100.00		
rifamycin		51.49	51.63				51.56	51.56	
tetracycline					33.81	33.81			

TABLE S7 Relative abundances of resistance classes across samples M1–M9.
